# MADS8 is indispensable for female reproductive development at high ambient temperatures in cereal crops

**DOI:** 10.1093/plcell/koad246

**Published:** 2023-09-21

**Authors:** Chaoqun Shen, Yueya Zhang, Gang Li, Jin Shi, Duoxiang Wang, Wanwan Zhu, Xiujuan Yang, Ludovico Dreni, Matthew R Tucker, Dabing Zhang

**Affiliations:** Joint International Research Laboratory of Metabolic and Developmental Sciences, State Key Laboratory of Hybrid Rice, School of Life Sciences and Biotechnology, Shanghai Jiao Tong University, Shanghai 20040, China; Waite Research Institute, School of Agriculture, Food and Wine, The University of Adelaide, Waite campus, Adelaide, South Australia 5064, Australia; Joint International Research Laboratory of Metabolic and Developmental Sciences, State Key Laboratory of Hybrid Rice, School of Life Sciences and Biotechnology, Shanghai Jiao Tong University, Shanghai 20040, China; Waite Research Institute, School of Agriculture, Food and Wine, The University of Adelaide, Waite campus, Adelaide, South Australia 5064, Australia; Joint International Research Laboratory of Metabolic and Developmental Sciences, State Key Laboratory of Hybrid Rice, School of Life Sciences and Biotechnology, Shanghai Jiao Tong University, Shanghai 20040, China; Joint International Research Laboratory of Metabolic and Developmental Sciences, State Key Laboratory of Hybrid Rice, School of Life Sciences and Biotechnology, Shanghai Jiao Tong University, Shanghai 20040, China; Joint International Research Laboratory of Metabolic and Developmental Sciences, State Key Laboratory of Hybrid Rice, School of Life Sciences and Biotechnology, Shanghai Jiao Tong University, Shanghai 20040, China; Waite Research Institute, School of Agriculture, Food and Wine, The University of Adelaide, Waite campus, Adelaide, South Australia 5064, Australia; Joint International Research Laboratory of Metabolic and Developmental Sciences, State Key Laboratory of Hybrid Rice, School of Life Sciences and Biotechnology, Shanghai Jiao Tong University, Shanghai 20040, China; Waite Research Institute, School of Agriculture, Food and Wine, The University of Adelaide, Waite campus, Adelaide, South Australia 5064, Australia; Joint International Research Laboratory of Metabolic and Developmental Sciences, State Key Laboratory of Hybrid Rice, School of Life Sciences and Biotechnology, Shanghai Jiao Tong University, Shanghai 20040, China; Waite Research Institute, School of Agriculture, Food and Wine, The University of Adelaide, Waite campus, Adelaide, South Australia 5064, Australia

## Abstract

Temperature is a major factor that regulates plant growth and phenotypic diversity. To ensure reproductive success at a range of temperatures, plants must maintain developmental stability of their sexual organs when exposed to temperature fluctuations. However, the mechanisms integrating plant floral organ development and temperature responses are largely unknown. Here, we generated barley and rice loss-of-function mutants in the SEPALLATA-like MADS-box gene *MADS8*. The mutants in both species form multiple carpels that lack ovules at high ambient temperatures. Tissue-specific markers revealed that HvMADS8 is required to maintain floral meristem determinacy and ovule initiation at high temperatures, and transcriptome analyses confirmed that temperature-dependent differentially expressed genes in *Hvmads8* mutants predominantly associate with floral organ and meristem regulation. HvMADS8 temperature-responsive activity relies on increased binding to promoters of downstream targets, as revealed by a cleavage under targets and tagmentation (CUT&Tag) analysis. We also demonstrate that HvMADS8 directly binds to 2 orthologs of D-class floral homeotic genes to activate their expression. Overall, our findings revealed a new, conserved role for MADS8 in maintaining pistil number and ovule initiation in cereal crops, extending the known function of plant MADS-box proteins in floral organ regulation.

## Teaser

MADS8 maintains pistil number and ovule initiation at high ambient temperature in barley and rice via a temperature-dependent increase in binding to its downstream targets.

**IN A NUTSHELL**

**Background:** Temperature is a major factor regulating plant growth and phenotypic diversity. When exposed to temperature fluctuations, plants must maintain the developmental stability of their sexual organs in order to survive and reproduce, especially in the face of current and expected global warming trends. In the grass family, the development of the 4 concentric flower whorls including the pistil, the innermost female reproductive organ, contributes directly to reproductive success. However, the genes and mechanisms maintaining floral organ identity and development in response to high temperature remain underexplored.

**Question:** We wanted to know how female reproductive development is maintained in cereal crops at high ambient temperatures, and whether members of the SEPALLATA subfamily of floral organ regulators contribute.

**Findings:** We discovered that barley (*Hordeum vulgare*) and rice (*Oryza sativa*) *MADS8* genes are indispensable for female reproductive development at high temperatures. Knockout mutants lacking functional MADS8 exhibited multiple pistils with no ovule at high ambient temperatures. We also showed that HvMADS8 increases its binding to the promoters of its downstream target genes in a temperature-responsive manner, which maintains floral meristem determinacy and ovule initiation at high temperatures. Our results shed light on the conserved molecular mechanism that stabilizes female reproductive development in grass species exposed to high temperatures, extending the known functions of floral organ identity regulators.

**Next steps:** We aim to stabilize and improve crop yields under high-temperature conditions. Further study will focus on understanding pathways regulating reproductive organ development in greater detail and engineering plants with improved developmental stability in the context of global warming by manipulating the expression of these key regulators.

## Introduction

To survive and reproduce, plants must adjust their physiology and development to the changing environment, especially in the face of current and expected global warming (Zhao et al. 2017; Vu et al. 2019). High, but not stressful, ambient temperatures induce a series of morphological adjustments in plants, collectively termed thermomorphogenesis, such as reduced leaf area and elongated hypocotyls (Franklin 2009; Casal and Balasubramanian 2019). Several thermosensors and thermally responsive elements, such as phytochrome B (phyB), PHYTOCHROME INTERACTING FACTOR 4 (PIF4), and the histone variant H2A.Z, have been identified in the model plant Arabidopsis (*Arabidopsis thaliana*) (Kumar and Wigge 2010; Quint et al. 2016; Ding et al. 2020; Xue et al. 2021).

In the past 2 decades, molecular genetic studies have uncovered genes essential for reproductive development at high temperatures in grasses (Poaceae). These studies have focused on rice (*Oryza sativa*), where thermosensitive genic male sterile (TGMS) lines that are male sterile at high temperatures but have normal fertility at low temperatures are widely used in breeding (Chen et al. 2007; Zhou et al. 2014; Yu et al. 2017; Zhu et al. 2020; Wu et al. 2022). Recently, a maize (*Zea mays*) thermosensitive mutant, *thermosensitive vanishing tassel1-R* (*tvt1-R*), was characterized (Xie et al. 2020). However, compared to the extensively studied regulators of vegetative growth, the genes and mechanisms maintaining floral organ identity and development in response to high temperature are underexplored.

Another economically important Poaceae crop, barley (*Hordeum vulgare*), has flowers with a structure similar to those of rice. Within each barley floret are 4 concentric whorls; the outermost whorl produces 2 bract-like organs (lemma and palea) that surround the inner 3 whorls, which produce the 2 lodicules, 3 stamens, and a pistil born from the 3 fused carpel primordia, respectively (Kellogg 2001; Dreni et al. 2013). The mature pistil, also referred to as the gynoecium, is the female reproductive organ and consists predominantly of a carpel that bears a single ovule. Carpels develop on the basis of timed floral meristem (FM) termination (Shen et al. 2021). Unlike indeterminate barley inflorescence meristems that can continuously produce lateral primordia, the FM is determinate, which means its stem cells will be consumed by the final floral organ initiated from it (Chu et al. 2006; Dreni et al. 2007; Yamaki et al. 2011). Several mutants with loss of FM determinacy displaying extra pistils have been reported in cereal plants (Yang and Tucker 2021). *FLORAL ORGAN NUMBER* (*FON*) genes in rice control FM size, and loss-of-function *fon* mutants produce larger meristems with supernumerary anthers and pistils that can produce grain (Jiang et al. 2005; Chu et al. 2006; Moon et al. 2006; Suzaki et al. 2006). Florets from tri-pistil mutants in wheat (*Triticum aestivum*) produce 3 pistils that occasionally contain multiple ovules and generate 1 to 3 grains in a single floret (Peng et al. 2008; Li et al. 2020; Liu et al. 2022). The 2 extra pistils appear to be initiated late in floret development, and anthers are retained, suggesting that the tri-pistil phenotype may relate to meristem determinacy (Nagasawa et al. 2003; Li et al. 2020; Selva et al. 2021; Liu et al. 2022).

According to the ABCDE combinatorial model proposed to explain flower development in Arabidopsis, snapdragon (*Antirrhinum majus*), and petunia (*Petunia hybrida*), C-class and D-class homeotic genes are required for carpel and ovule development, while E-class genes are essential for the function of the other 4 classes (Colombo et al. 1995; Pelaz et al. 2000, 2001; Favaro et al. 2003; Pinyopich et al. 2003). Except for the A-class *APETALA 2* (*AP2*) gene, all ABCDE genes encode MIKC-type MADS-box transcription factors (Jofuku et al. 1994). These proteins bind as dimers to DNA sequences named “CArG” boxes and interact to form heterotetrameric complexes that can recognize different CArG targets (Muiño et al. 2014; Gusewski et al. 2017). In rice, there are 2 duplicated D-lineage genes, *OsMADS13* and *OsMADS21*; *Osmads13* mutants result in the homeotic conversion of ovules into carpels (Dreni et al. 2007). Furthermore, reiterative carpel development in *Osmads13* lines indicates a role for OsMADS13 in FM determinacy, confirmed by the prolonged expression of the meristematic cell marker *ORYZA SATIVA HOMEOBOX 1* (*OSH1*), in *Osmads13* florets (Yamaki et al. 2011). Recent transcriptome analysis revealed the upregulation of 3 rice carpel identity genes in *Osmads13* FM and ovule primordia, namely *DROOPING LEAF* (*DL*) and the 2 C-class genes *OsMADS3* and *OsMADS58*, indicating an important repressor role for *OsMADS13* in carpel development (Osnato et al. 2021). In contrast, OsMADS21 appears to play no crucial role in the formation of reproductive organs (Dreni et al. 2007). Nevertheless, the *Osmads13* phenotype can still be partially rescued by *OsMADS21* expressed from the *OsMADS13* promoter, suggesting it has retained some ovule identity determination activity (Dreni et al. 2011).

E-class proteins are encoded by *SEPALLATA* (*SEP*) subfamily MADS-box genes, which can be further divided into 2 subclades in all angiosperms: in rice, the *LOFSEP* clade contains *OsMADS1*, *OsMADS5*, and *OsMADS34*, while the *SEP3* clade comprises *OsMADS7* and *OsMADS8* (also known as *OsMADS45* and *OsMADS24*, respectively) (Malcomber and Kellogg 2005; Zahn et al. 2005; Arora et al. 2007). All rice *SEP* genes have a conserved and redundant role in pistil development. Pistils are either increased or completely missing in several combinations of mutants defective in *LOFSEP* clade members (Wu et al. 2018), and the simultaneous knockdown of *OsMADS7* and *OsMADS8* causes different degrees of defects in the inner 3 whorls, affecting carpel fusion and FM determinacy with additional reproductive organ-like structures initiated inside the mutant carpels (Cui et al. 2010).

HvMADS1 was recently shown to be responsible for maintaining high temperature-responsive inflorescence meristem determinacy in barley (Li et al. 2021). At high temperatures, HvMADS1 exhibits increased binding to A-tract CArG-box promoter elements in downstream target genes to regulate inflorescence differentiation and phytohormone signaling, thereby integrating temperature responses and cytokinin homeostasis to repress meristem activity. This research extended the role of SEP proteins in inflorescence thermomorphogenesis while raising more questions as to whether and how other SEP proteins are involved in thermally responsive development.

In this study, we demonstrate the conserved role of MADS8 in regulating the formation of a single pistil at high ambient temperature in 2 grass species, barley and rice. In their respective *mads8*-null mutants, floral organs develop normally under control temperatures, but extra carpels repeatedly form without ovules at high temperatures. Our results demonstrate that HvMADS8 is crucial in maintaining FM determinacy and ovule initiation under high ambient temperature by modulating the expression of downstream genes in flower development pathways in a temperature-dependent manner. Furthermore, our study offers new understanding and genetic materials that might be used in the context of managing plant responses to global warming.

## Results

### HvMADS8 regulates pistil number in response to high temperature

**Figure 1.**
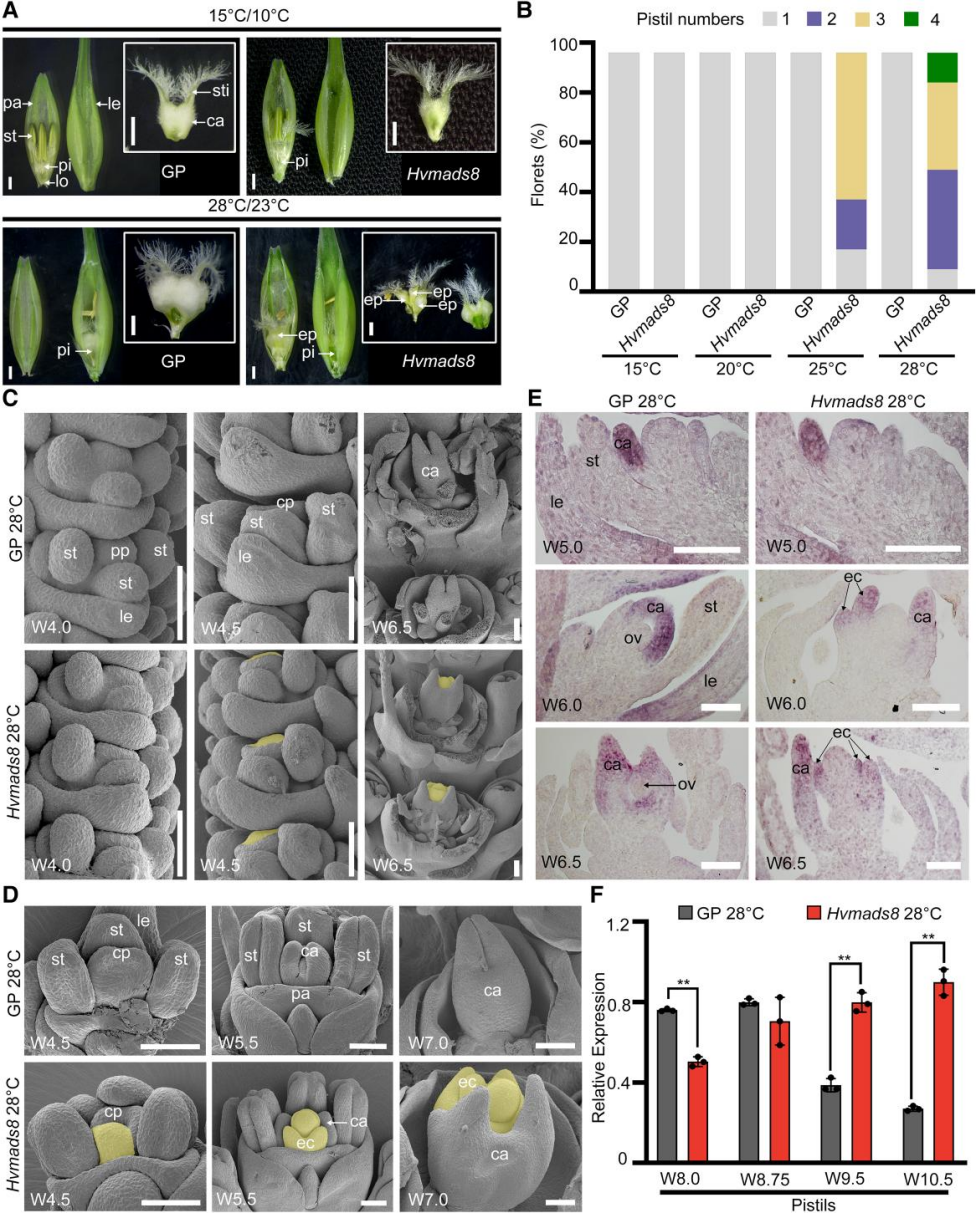
HvMADS8 regulates pistil number in response to high temperature. **A)** Pistil phenotypes of the WT GP and *Hvmads8* mutants grown under control (15 °C day/10 °C night) or high (28 °C day/23 °C night)-temperature conditions. ca, carpel; ep, extra pistil; le, lemma; lo, lodicule; pa, palea; pi, pistil; st, stamen; sti, stigma. Scale bars, 1 mm. **B)** Pistil number in GP and *Hvmads8* florets grown at different temperatures. **C, D)** SEM of GP and *Hvmads8* spike **C)** and floret **D)** at 28 °C at different developmental stages. cp, carpel primordia; ec, extra carpel; pp, pistil primordia. Extra carpel primordia bulges and extra carpels are shaded in yellow. Scale bars, 100 *μ*m. **E)** In situ hybridization showing the expression of the carpel marker gene, *HvDL*, in GP and *Hvmads8* florets at 28 °C. ov, ovule. Scale bars, 100 *μ*m. **F)** Relative expression of *HvDL* as determined by RT-qPCR at different stages in GP and *Hvmads8* pistils at 28 °C, relative to *HvActin7*. Values are means ± Sd; *n* = 3 biological replicates. Asterisks indicate significant differences (2-way ANOVA test; **P < 0.01).

Having previously shown that spikes of *Hvmads1* mutants (*LOFSEP* clade) produce ectopic spike/spikelet-like organs at high temperatures (Li et al. 2021), we examined the inflorescence phenotype of a *SEP3* clade mutant at different temperature conditions. Using clustered regularly interspaced short palindromic repeat (CRISPR)/CRISPR-associated nuclease 9 (Cas9)-mediated gene editing (Ma et al. 2015), we generated loss-of-function *Hvmads8* mutants in the commonly used UK barley variety Golden Promise (GP) and the Australian barley variety WI4330 (WI), which is adapted to a warmer climate (Supplemental Fig. S1A). Under control temperatures (15 °C day/10 °C night), the *Hvmads8* mutant exhibited no visible changes in flower organ morphology in either background (Fig. 1A; Supplemental Fig. S1B). However, at high temperatures (28 °C day/23 °C night), the mutant lines produced extra pistil-like structures in the fourth (innermost) whorl in the GP and WI backgrounds, while other floral organs maintained a wild-type (WT) structure (Fig. 1A; Supplemental Fig. S1B).

To confirm this temperature-associated phenotype, we grew WT GP and mutant plants at various temperatures, with control (15 °C) and higher ambient temperatures (20, 25, and 28 °C). Accordingly, we grew seedlings to Waddington stage 1 (W1, inflorescence meristem initiation; Waddington et al. 1983) at 15 °C before subjecting them to high-temperature treatment. After heat treatment, we dissected W9.5 (prior to pollination) florets from WT and *Hvmads8* lines and analyzed pistil number. The *Hvmads8* lines developed normal pistils with no detectable difference in seed setting rates or grain morphologies compared to WT at 15 or 20 °C (Fig. 1B; Supplemental Fig. S1, C and D). At 25 °C, only 18% (17, *n* = 96) of the flowers produced 1 pistil, while other flowers had 2 or 3 pistil-like structures. When temperatures increased to 28 °C, the maximum number of pistil-like structures per floret increased to 4, and the proportion of flowers with 1 pistil decreased to 9% (9, *n* = 96; Fig. 1B). Detailed phenotypic analysis revealed partial extra carpel structures such as stigmas within the single pistil with no distinguishable ovule structure within the ovary (Supplemental Fig. S1E). Consequently, *Hvmads8* formed no mature grains at either 25 or 28 °C (Supplemental Fig. S1F). Overall, the number of extra pistils increased with temperature, indicating a dosage, but not a threshold, effect of temperature on the *Hvmads8* phenotype, similar to *Hvmads1*.

We turned to scanning electron microscopy (SEM) to trace pistil development, particularly in the context of carpel growth and differentiation during early stages of inflorescence and floret development. In WT florets, pistil development begins with the initiation of pistil primordia at W4.0 (Fig. 1C). At W4.5, a bulge corresponding to the compound carpel primordium arises first on the lemma side of the FM (Fig. 1, C and D). At stages W5 to W5.5, the carpel primordia elongate to enclose the developing ovule primordium (Fig. 1D). In early-stage *Hvmads8* inflorescences, the first pistil and carpel primordia initiated as in WT at W4.0 and W4.5; however, the FM in the center of the gynecium started to display abnormal expansion toward the palea side at W4.5 (Fig. 1, C and D). After W5.0, when the WT ovule primordium is enclosed by the fused compound carpel, extra carpels repeatedly initiated inside the *Hvmads8* gynecium, with the innermost carpel maintaining an abnormal FM-like bulge even at W7.0 (Fig. 1D).

Using the barley ortholog of the rice carpel marker gene *DL* as a probe, we performed in situ hybridization to confirm the identities of extra *Hvmads8* carpels. As in rice, the barley *HvDL* gene is specifically expressed in the lemma and carpel primordia in WT (Fig. 1E) (Yamaguchi et al. 2004). In the first initiated *Hvmads8* carpel, we observed no significant changes in *HvDL* expression relative to WT (W5.0; Fig. 1E). However, at later stages, when the WT ovule primordium has already been enveloped by the carpel, we detected ectopic *HvDL* transcripts in extra *Hvmads8* carpel primordia growing inside the first initiated carpels (Fig. 1E). We detected an overall increased level of *HvDL* transcripts in late-stage *Hvmads8* mutant pistils (W10.5, stage after pollination) grown at high temperatures, in contrast the decreasing *HvDL* transcript levels seen in WT after completion of pistil development (Fig. 1F). Collectively, these results demonstrate that repeated carpel initiation occurs inside the previously formed carpels throughout gynecium development in *Hvmads8* lines, indicating a deficiency in FM determinacy and ovule development.

### HvMADS8 induces ovule initiation and establishes FM determinacy

**Figure 2.**
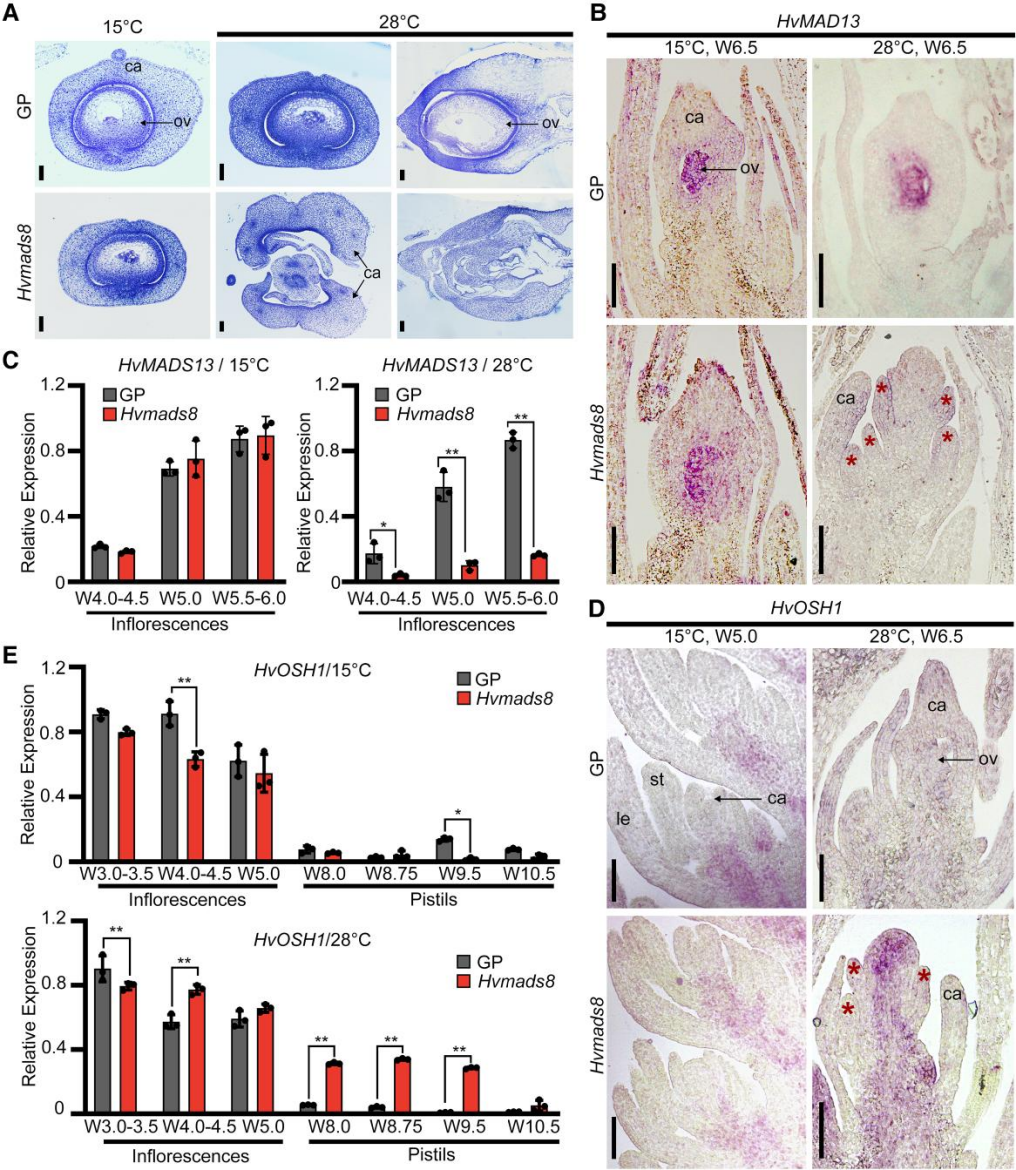
HvMADS8 induces ovule initiation and establishes FM determinacy. **A)** Semi-thin sections of W9.5 GP and *Hvmads8* pistils at 15 °C (horizontal sections) or 28 °C (left, horizontal sections; right, longitudinal sections). ca, carpel; ov, ovule. Scale bars, 100 *μ*m. **B)** In situ hybridization showing expression of *HvMADS13* (ovule marker gene) in GP and *Hvmads8* florets at 15 and 28 °C. Asterisks indicate extra carpels in *Hvmads8* lines at 28 °C. Scale bars, 100 *μ*m. **C)** Relative expression of *HvMADS13* at early stages of inflorescence development at 15 and 28 °C. Values are means ± Sd; *n* = 3 biological replicates. Asterisks indicate significant differences (2-way ANOVA test; *P < 0.05, **P < 0.01). **D)** In situ hybridization showing expression of *HvOSH1* (meristematic cell marker gene) in GP and *Hvmads8* inflorescences and florets at 15 and 28 °C. le, lemma; st, stamen. Asterisks indicate extra carpels in *Hvmads8* lines at 28 °C. Scale bars, 100 *μ*m. **E)** Relative expression of *HvOSH1* at different stages of inflorescence and pistil development at 15 and 28 °C. Values are means ± Sd; *n* = 3 biological replicates. Asterisks indicate significant differences (2-way ANOVA test; *P < 0.05, **P < 0.01).

To further investigate *Hvmads8* ovule development, we performed a detailed histological analysis using W9.5 florets. In WT W9.5 pistils, the fully mature ovule is composed of 3 tissues: the outer and inner integuments, nucellus, and embryo sac (Fig. 2A). At control temperatures, we observed the complete ovule structure in *Hvmads8* pistils, whereas *Hvmads8* pistils grown at high temperatures lacked distinguishable ovule tissues inside them (Fig. 2A). We used *HvMADS13*, the barley homolog of the rice ovule identity gene *OsMADS13*, to further elucidate ovule initiation status. In WT, *HvMADS13* expression mirrored that of *OsMADS13* in rice, which is highly specific to the developing ovule and inner layer of the carpel that develops into the ovary wall (Fig. 2B) (Dreni et al. 2007, 2011). *HvMADS13* expression in *Hvmads8* pistils at 15 °C resembled WT but at 28 °C, *HvMADS13* expression was missing from the center of the pistil, with only weak expression detected in carpels (Fig. 2B). Reverse transcription quantitative PCR (RT-qPCR) analysis confirmed significantly decreased *HvMADS13* expression levels in early-stage W4.0 to 6.0 inflorescences at high temperatures (Fig. 2C). Taken together, these data show that ovule initiation is severely disrupted in *Hvmads8* lines at high temperatures, resulting in the disappearance of all ovule structures.

In the center of *Hvmads8* carpels, ovules are repeatedly replaced by a new, indeterminate carpel-like tissue; this phenotype suggests that floral stem cells are not terminated correctly. The barley homolog of the rice FM marker gene *OSH1* exhibited ectopic and prolonged expression in *Hvmads8* carpels (Fig. 2, D and E). In situ hybridization analysis revealed that *HvOSH1* continues to be expressed in the central region after several extra *Hvmads8* carpels formed at high temperatures, whereas its expression disappeared from the flower center after carpel formation in WT and *Hvmads8* florets at control temperatures (Fig. 2D). We confirmed this prolonged expression of *HvOSH1* by RT-qPCR analysis, which revealed transcript levels up to 10-fold higher in late-stage (W8.0 to 10.5) mutant pistils collected at 28 °C (Fig. 2E). Notably, *HvMADS13* expression (Fig. 2B) was completely missing from the expanded expression zone of *HvOSH1* (Fig. 2D), suggesting a close link between ovule initiation and FM determinacy. Based on these results, we conclude that HvMADS8 functions to induce ovule initiation and establish meristem determinacy in the center of the FM, as loss of HvMADS8 activity leads to missing ovule structures and FM indeterminacy.

### Temporal and spatial expression patterns of *HvMADS8* at different temperatures

**Figure 3.**
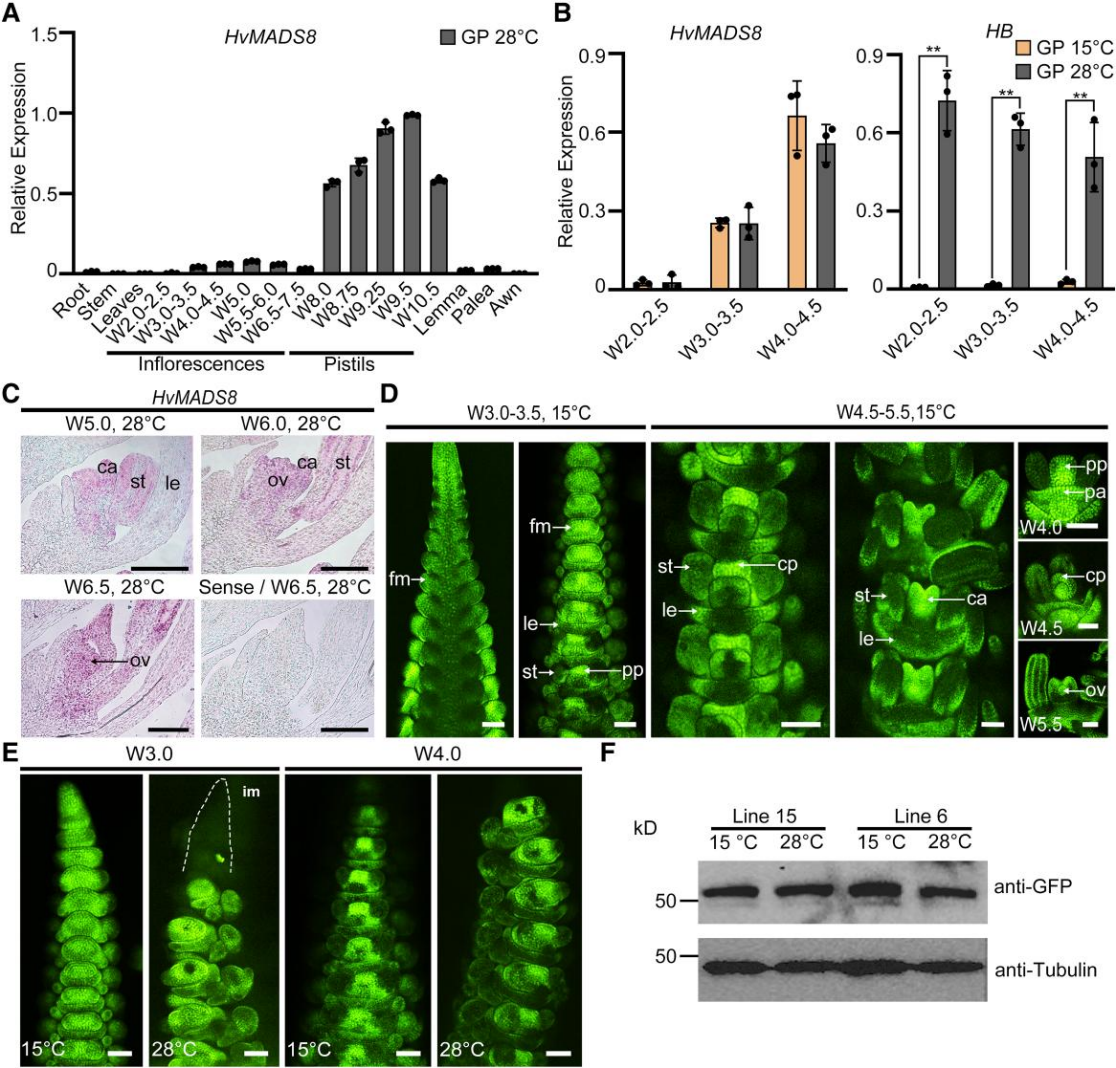
Temporal and spatial expression of *HvMADS8* at different temperatures. **A)** Relative expression of *HvMADS8* in different GP plant tissues and at different stages of development at 28 °C. Values are means ± Sd; *n* = 3 biological replicates. **B)** Relative expression of *HvMADS8* at early stages of inflorescence development at 15 and 28 °C. Expression of *HB* (temperature-responsive gene) was used as positive control. Values are means ± Sd; *n* = 3 biological replicates. Asterisks indicate significant differences (2-way ANOVA test; **P < 0.01). **C)** In situ hybridization showing expression of *HvMADS8* at stages W5.0 to 6.5 at 28 °C in longitudinal sections of GP florets. The sense probe served as a negative control. ca, carpel; le, lemma; ov, ovule; st, stamen. Scale bars, 100 *μ*m. **D)** Accumulation of HvMADS8 in spikes and florets from W3.0 to 5.5 in *proHvMADS8:HvMADS8-eGFP* transgenic lines at 15 °C. cp, carpel primordia; fm, floral meristem; pa, palea; pp, pistil primordia. Scale bars, 100 *μ*m. **E)** Accumulation of HvMADS8 in W3.0 and W4.0 *proHvMADS8:HvMADS8-eGFP* transgenic spikes grown at 15 and 28 °C. im, inflorescence meristem. Scale bars, 100 *μ*m. **F)** Immunoblot analysis of HvMADS8-eGFP abundance in W5.5 to 6.5 spikes from 2 independent *proHvMADS8:HvMADS8-eGFP* lines grown at 15 and 28 °C. Tubulin served as loading control. All experiments were independently performed at least 3 times with similar results.

To elucidate how HvMADS8 regulates temperature-responsive pistil development, we analyzed its temporal and spatial expression pattern throughout flower development. We first detected *HvMADS8* transcripts in inflorescence meristems from W3.0 to 3.5 (lemma and stamen primordia formation), accumulating later in carpels and ovules (Fig. 3, A and C). Strong transcript accumulation was subsequently detected in maturing pistils, which were dissected from florets from W8.0 onwards (Fig. 3A). Using the temperature-responsive gene *Homeobox* (*HB*) as a positive control for high temperature responses (Li et al. 2021), *HvMADS8* expression at different temperatures was not significantly altered in early inflorescence meristems (W2.0 to 4.5; Fig. 3B). Similarly, HvMADS8 protein in *proHvMADS8:HvMADS8*-enhanced GFP (*eGFP*) transgenic lines showed a broad distribution in early-stage florets and enriched accumulation in pistils after the formation of pistil primordia at control temperatures (Fig. 3D), which did not change in distribution or amount at high temperatures (Fig. 3, E and F). Thus, neither HvMADS8 mRNA nor protein levels are affected by temperature during early development (W2.0 to W6.5).

### HvMADS8 coordinates thermal transcriptome programming of floral development

**Figure 4.**
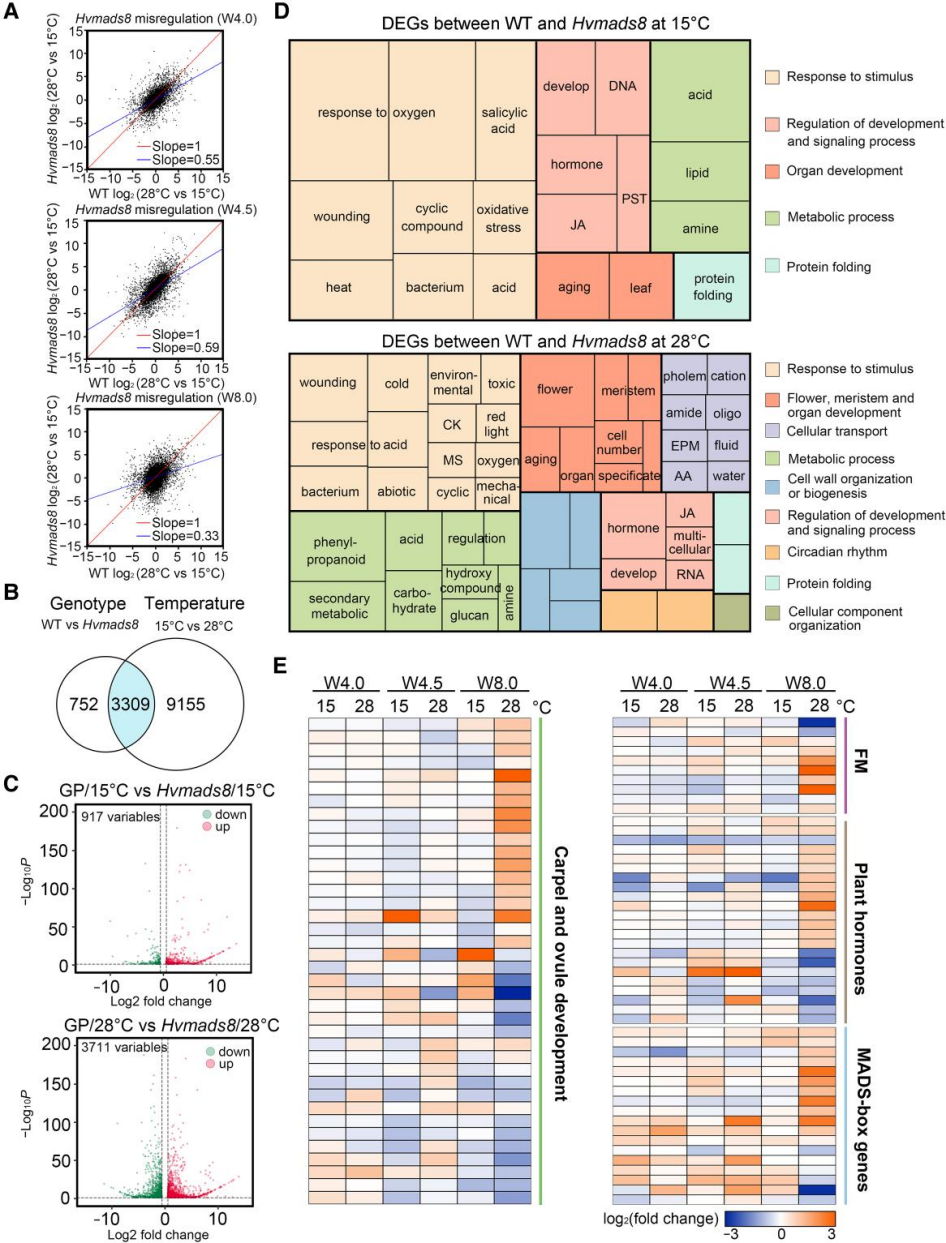
HvMADS8 coordinates the thermal transcriptome programming of floral development. **A)** Correlation analyses of transcript levels at control and high temperatures at 3 stages of *Hvmads8* and GP carpel development (W4.0, W4.5, and W8.0). **B)** Overlap of 13,216 DEGs responding to genotype and temperature. The blue-shaded region contains genes coregulated by genotype and temperature. **C)** Volcano plots showing differential gene expression in GP relative to *Hvmads8* at 15 and 28 °C at the W4.0, W4.5, and W8.0 stages of inflorescence and carpel development. **D)** GO term enrichment analysis of DEGs between WT and *Hvmads8* at 15 and 28 °C. CK, response to cytokinin; JA, regulation of jasmonic acid–mediated signaling; MS, response to monosaccharides; PST, phosphorelay signal transduction; EPM, export across the plasma membrane; AA, amino acid transport. **E)** Heatmap representation of expression levels for DEGs involved in carpel and ovule development, or FM regulation; plant hormone regulation; or that encode MADS-box proteins.

We compared the transcriptome patterns (transcriptome deep sequencing [RNA-seq]) of WT and *Hvmads8* lines grown at 28 and 15 °C to examine downstream biological pathways or gene regulation programs influenced by the *Hvmads8* mutation. The 3 stages analyzed covered early inflorescence meristems before and after the first extra carpel primordia bulge appears (stages W4.0 and W4.5), and developing pistils at W8.0 dissected (1 mm in length) from the young inflorescence. Correlation analyses using global transcriptional patterns at these stages revealed significant dysregulation of the expression of thermal response genes between WT and *Hvmads8* lines (Fig. 4A). We identified 13,216 differentially expressed genes (DEGs) in total, with genes affected by genotype (WT compared to *Hvmads8*) or temperature (15 °C compared to 28 °C; Fig. 4B; Supplemental Data Set 1). Of the 4,061 DEGs responding to genotype, 3,309 (81.5%) were also affected by temperature (Fig. 4B). Changes in the expression of DEGs were much greater at 28 °C (Fig. 4C), highlighting the essential role of HvMADS8 in regulating gene expression at higher temperatures.

Gene ontology (GO) analysis revealed that compared to 15 °C, DEGs at high temperatures are largely associated with flower development and meristem fate, and also enriched for cellular transport and cell wall biogenesis, possibly due to the extra carpel growth in *Hvmads8* florets (Fig. 4D; Supplemental Data Set 2). Consistent with the clear FM and ovule defects in high temperature-grown *Hvmads8* lines, genes encoding MADS-box proteins and barley homologs of rice and Arabidopsis proteins involved in carpel and ovule development, FM regulation, and plant hormone pathways were overrepresented among the DEGs at 28 °C (Fig. 4E; Supplemental Data Set 3). For example, homologs of *YABBY*, *AINTEGUMENTA* (*ANT*), and *SHI-RELATED SEQUENCE* (*SRS1*) genes, which are all involved in the development of Arabidopsis carpel and ovules, displayed expression changes in *Hvmads8* tissues at elevated temperature (Fig. 4E; Supplemental Fig. S2, A to C). Moreover, genes encoding meristem regulators such as CLAVATA-like, KNOX-like, and HvLFY (LEAFY) generally exhibited higher expression in *Hvmads8* tissues, especially in pistils at 28 °C (Fig. 4E; Supplemental Fig. S2, D to F), supporting the observed changes in FM termination (Fig. 2, B and D).

Prominent expression changes in *Hvmads8* mutants occurred in the genes encoding MADS-box proteins. C-class (*HvMADS3* and *HvMADS58*) and E-class (*HvMADS1*) genes were upregulated at 28 °C (Supplemental Fig. S2, G to I). As the E-class proteins have been reported to upregulate C-class genes (Kaufmann et al. 2009; Wu et al. 2018), these expression changes of the 2 C-class members in *Hvmads8* may be caused by an indirect effect due to the increased expression of *HvMADS1* at W8.0. D-class (*HvMADS13* and *HvMADS21*) and B-sister class (*HvMADS31*) genes were downregulated (Figs. 4E and 2C; Supplemental Fig. S2, J to L), suggesting that other MADS-box genes in the ABCDE model may contribute to extra pistil formation in the *Hvmads8* mutant. In addition, the barley homologs of many thermal response genes, including those encoding heat shock proteins, showed significant changes (increases or decreases) in their expression based on genotype and/or temperature (Fig. 4A; Supplemental Fig. S2M). We conclude that HvMADS8 has a critical regulatory role in thermal transcriptome programming at high temperatures, especially in terms of flower and meristem regulation.

### Increased HvMADS8 binding activity contributes to floral development transcriptome reprogramming

Stable *HvMADS8* transcript and HvMADS8 protein levels at high temperatures (Fig. 3) were reminiscent of HvMADS1 under similar conditions; hence, we speculated that HvMADS8 may function in a similar manner as HvMADS1 (Li et al. 2021), through temperature-dependent changes in binding affinity to regulate downstream gene expression. We therefore used W4.5 inflorescence meristems from a *proHvMADS8:HvMADS8*-*eGFP* transgenic line to perform cleavage under targets and tagmentation (CUT&Tag) analyses to identify binding targets of HvMADS8 at low and high temperatures.

**Figure 5.**
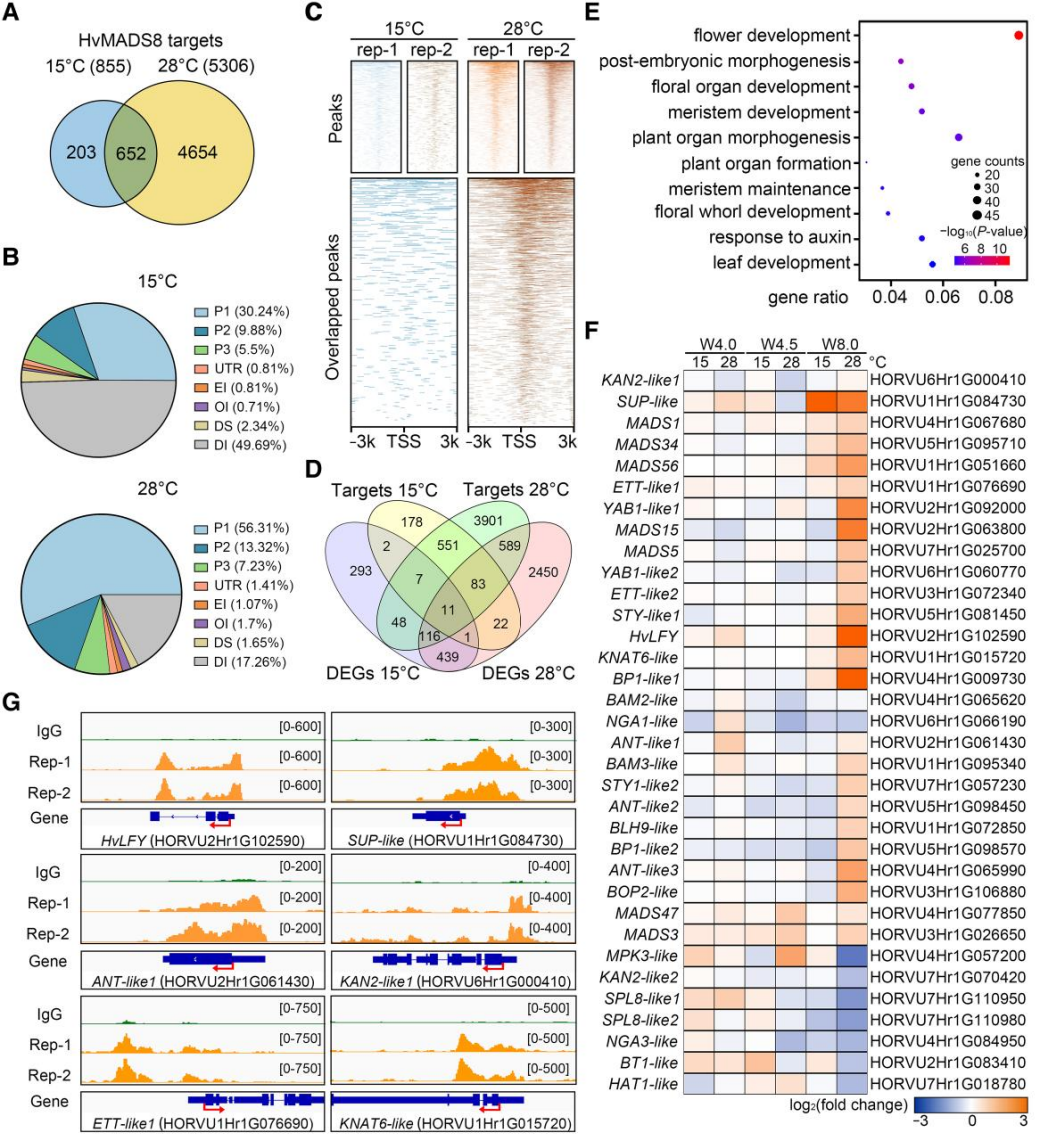
Changes in HvMADS8 binding activity at high temperatures contribute to transcriptome reprogramming for floral development. **A)** Venn diagram showing the numbers and overlap of target genes bound by HvMADS8 at 15 and 28 °C. **B)** Distribution of HvMADS8-bound regions in different barley genomic regions. P1, promoters (<1 kb upstream of ATG); P2, promoters (1 to 2 kb upstream of ATG); P3, promoters (2 to 3 kb upstream of ATG); UTR, 3′ untranslated region; EI, exons and first intron; OI, other introns; DS, downstream; DI, distal intergenic. **C)** Density heatmaps showing CUT&Tag peaks upstream and downstream of the TSS at 15 and 28 °C. **D)** Venn diagram showing the overlap of HvMADS8-regulated DEGs and binding targets at 15 and 28 °C. **E)** GO term enrichment analysis of 799 genes common to DEGs and CUT&Tag targets at 28 °C. **F)** Heatmap representation of the expression of HvMADS8-bound DEGs involved in pistil and FM development. **G)** Chromatin-binding profiles of HvMADS8 in the promoters of 6 selected pistil regulation genes. CUT&Tag reads of 2 GFP biological repeats (Rep-1 and Rep-2; orange) and the IgG negative control (green) were visualized using the Integrative Genomics Viewer. Arrows in the gene model indicate transcription start sites, the larger boxes indicate exons, the smaller boxes illustrate untranslated regions combined from all alternative transcripts, and the lines indicate introns.

We identified far more HvMADS8 binding targets at high compared to low temperatures (5,306 and 855, respectively; Fig. 5A; Supplemental Data Set 5). Approximately 76% (652/855) of the targets identified at 15 °C were in the 28 °C target set (Fig. 5A), suggesting that an increase, rather than a switch, in HvMADS8 targets occurred at higher temperatures. Consistent with HvMADS8 functioning as a transcription factor, 46% of the total binding peaks were located <3 kb upstream of gene transcriptional start sites at 15 °C, a percentage that increased to 77% at 28 °C; 56% of binding sites were within 1 kb of the transcriptional start sites (Fig. 5B). Furthermore, the HvMADS8 binding region sites from 2 biological replicates were highly enriched around the transcription start sites at 28 °C, while the binding sites at 15 °C did not show this pattern (Fig. 5C). These data collectively indicate that HvMADS8 regulates temperature-responsive gene expression through differential binding to promoters of downstream targets.

Integrating DEG (RNA-seq) and binding target (CUT&Tag) data revealed 21 and 799 DEGs at 15 and 28 °C, respectively, that are bound by HvMADS8, which represent 2% and 15% of all HvMADS8 binding targets (Fig. 5D). GO analysis of the 799 gene targets at 28 °C revealed that 8 of the top 10 categories affect flower and meristem development or plant organ morphogenesis (Fig. 5E). Among these genes, we noticed a large number of the carpel, ovule, and FM regulators (Fig. 5F). Interestingly, the recently reported barley *HvLFY* gene showed a significant expression increase in *Hvmads8* W8.0 pistils, with binding peaks in its promoter and second intron, as visualized by the Integrative Genomics Viewer (Fig. 5, F and G) (Robinson et al. 2011). Given that HvLFY controls barley floral organ specification in early stages, upregulation of *HvLFY* may further indicate the overactivation of FMs in *Hvmads8* at higher temperatures (Selva et al. 2021). We also identified genes homologous to Arabidopsis *SUPERMAN* (*SUP*, which acts in the boundary specification between stamens in whorl 3 and carpels in whorl 4), *ANT*, and *KANADI 2* (*KAN2*, which functions in Arabidopsis gynecium patterning) as DEGs with obvious binding peaks in their promoter regions at 28 °C (Fig. 5, F and G) (Cucinotta et al. 2014; Prunet et al. 2017; Wils and Kaufmann 2017). Moreover, homologs to Arabidopsis meristem regulators *ETTIN* (*ETT*) and *KNOTTED1-LIKE HOMEOBOX GENE 6* (*KNAT6*), which function in ovule and gynecium development, both displayed direct binding by HvMADS8 (Fig. 5, F and G) (Ferrándiz et al. 2010; Kelley et al. 2012). Taken together, these data suggest that HvMADS8 affects transcriptome reprogramming through binding affinity changes that coordinate floral developmental pathways to inhibit the overactivation of FMs and stabilize female reproductive organ development at high temperatures.

### HvMADS8 directs D-class genes at high temperature

*OsMADS13* affects ovule initiation and FM termination (Dreni et al. 2007), and expression of the D-class genes *HvMADS13* and *HvMADS21* is affected by the *Hvmads8* mutation (Fig. 2C; Supplemental Fig. S2, J and K), prompting us to examine how these genes might interact to control ovule establishment in response to temperature.

**Figure 6.**
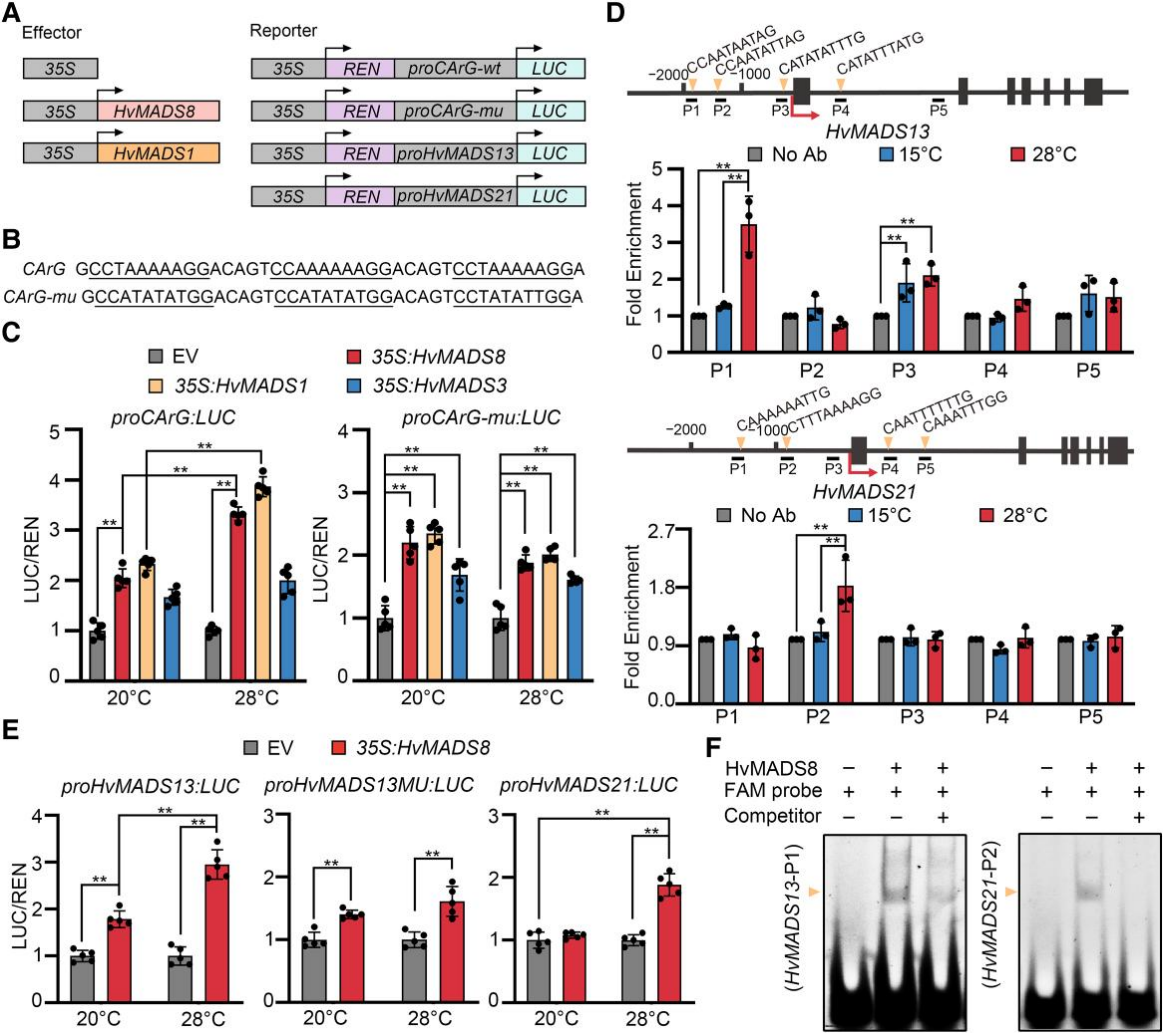
HvMADS8 regulates D-class genes at high temperatures. **A)** Diagrams of effector and reporter constructs for dual-LUC assays. The *HvMADS8* and *HvMADS1* effector genes were under the control of the 35S promoter. An empty 35S effector construct was used as a negative control. *LUC*, firefly luciferase; *REN*, *Renilla* luciferase. **B)** Artificial CArG-boxes with A-tracts (underlined, *CArG*, WT) or non-A-tracts (underlined, *CArG-mu*, mutant) used to drive *LUC* expression. **C)** Normalized luciferase (LUC/REN) activity derived from the artificial CArG-box promoters in the presence of HvMADS8, HvMADS1, HvMADS3, or empty vector (EV, negative control) at 20 and 28 °C. Values are means ± Sd; *n* = 5 biological replicates. Asterisks indicate significant differences (2-way ANOVA test; **P < 0.01). **D)** In vivo binding of *HvMADS13* and *HvMADS21* CArG-boxes by HvMADS8 at 15 and 28 °C, as determined by ChIP-qPCR. Upper, *HvMADS13* or *HvMADS21* genomic regions, showing locations of 5 DNA fragments (P1 to P5) for ChIP-qPCR. Boxes indicate exons; lines indicate promoter or intron regions; arrows indicate transcription start sites. Lower, fold enrichment of 5 DNA fragments at 15 or 28 °C relative to no-antibody (Ab) control. Values are means ± Sd; *n* = 3 biological replicates. Asterisks indicate significant differences (2-way ANOVA test; **P < 0.01). **E)** Normalized luciferase activity driven by the *HvMADS13* and *HvMADS21* promoters in the presence of HvMADS8 or EV (negative control) at 20 and 28 °C. The CArG-box in P1 binding site of the *HvMADS13* promoter is mutated to ATGTGAATAG in the *proHvMADS13MU:LUC* construct. Values are means ± Sd; *n* = 5 biological replicates. Asterisks indicate significant differences (2-way ANOVA test; **P < 0.01). **F)** EMSAs to detect interactions between HvMADS8 and the P1 (CArG-box motif of the *HvMADS13* promoter) and P2 (CArG-box motif of the *HvMADS21* promoter) probes.

Using a dual-luciferase transient expression assay in *Nicotiana benthamiana* leaves (Fig. 6A), we tested the ability of HvMADS8 to activate gene expression at 2 temperatures, 20 °C as a control, which is a normal growth temperature for *N. benthamiana* and also a relatively low temperature for *Hvmads8* with no obvious phenotypes shown (Fig. 1B), and 28 °C as the high temperature. Compared to the control, HvMADS3, which was previously shown to be temperature independent in regulating gene expression (Li et al. 2021), HvMADS8 exhibited temperature-dependent transcriptional activation only on artificial promoters carrying A-tract CArG-box motifs similar to HvMADS1 (Fig. 6, B and C). Using the inflorescence meristems of *proHvMADS8:HvMADS8*-*eGFP* plants grown at 15 and 28 °C, we investigated in vivo HvMADS8 binding targets by chromatin immunoprecipitation (ChIP) assays. HvMADS8 bound to promoter fragments harboring CArG-box sequences (primer set P1, P3 for *HvMADS13*; P2 for *HvMADS21*) of these 2 D-class genes, with significantly increased binding at high temperature (Fig. 6D). Transient luciferase expression assays also revealed that HvMADS8 activates the expression of *HvMADS13* and *HvMADS21* directly in a temperature-dependent manner, and the P1 motif of *HvMADS13* promoter contributed directly to this temperature-responsive activation (Fig. 6E). We confirmed the binding to P1 in the *HvMADS13* promoters and P2 in the *HvMADS21* promoter by electrophoretic mobility shift assays (EMSAs). We observed clearly shifted bands when HvMADS8 was incubated with CArG-box motif-containing probes labeled by fluorescein amidite (FAM); moreover, the band intensity decreased when unlabeled competitor probes were added (Fig. 6F). Collectively, these results demonstrate that HvMADS8 can bind to the promoters of the 2 barley D-class genes directly to regulate their expression, especially at high temperatures, which may prevent FM indeterminacy and overgrowth of carpels in WT plants.

### Physical interactions of HvMADS8 with other floral homeotic proteins

As the identity of different floral organs is determined by combinations of floral homeotic MADS-box proteins in Arabidopsis and rice (Cui et al. 2010; Theissen et al. 2016; Wu et al. 2018), we tested the interactions of HvMADS8 with other barley homologs of homeotic proteins under different temperatures using a yeast 2-hybrid assay (Supplemental Fig. S3). We used 15 °C as the lowest temperature, and 34 °C as the highest, since yeast cotransformants will not grow normally on synthetic-defined (SD) medium lacking Trp and Leu (SD/–Leu/–Trp [SD–2]) at 36 °C (Xie et al. 2020). HvMADS8 formed homodimers and heterodimers with all C-class, D-class, and E-class proteins except with HvMADS34 and the AGAMOUS-LIKE 6 (AGL6)-like protein HvMADS6 on selective medium (SD/–Ade/–His/–Leu/–Trp [SD–4]). Most of these interactions were insensitive to temperature, except for HvMADS21, HvMADS1, HvMADS5, and HvMADS6, which showed stronger interactions at 20 to 30 °C (Supplemental Fig. S3). These results suggest that HvMADS8 can form protein complexes with other floral homeotic proteins, most of which were not affected by temperature.

### Conserved OsMADS8 function in temperature-dependent pistil regulation

**Figure 7.**
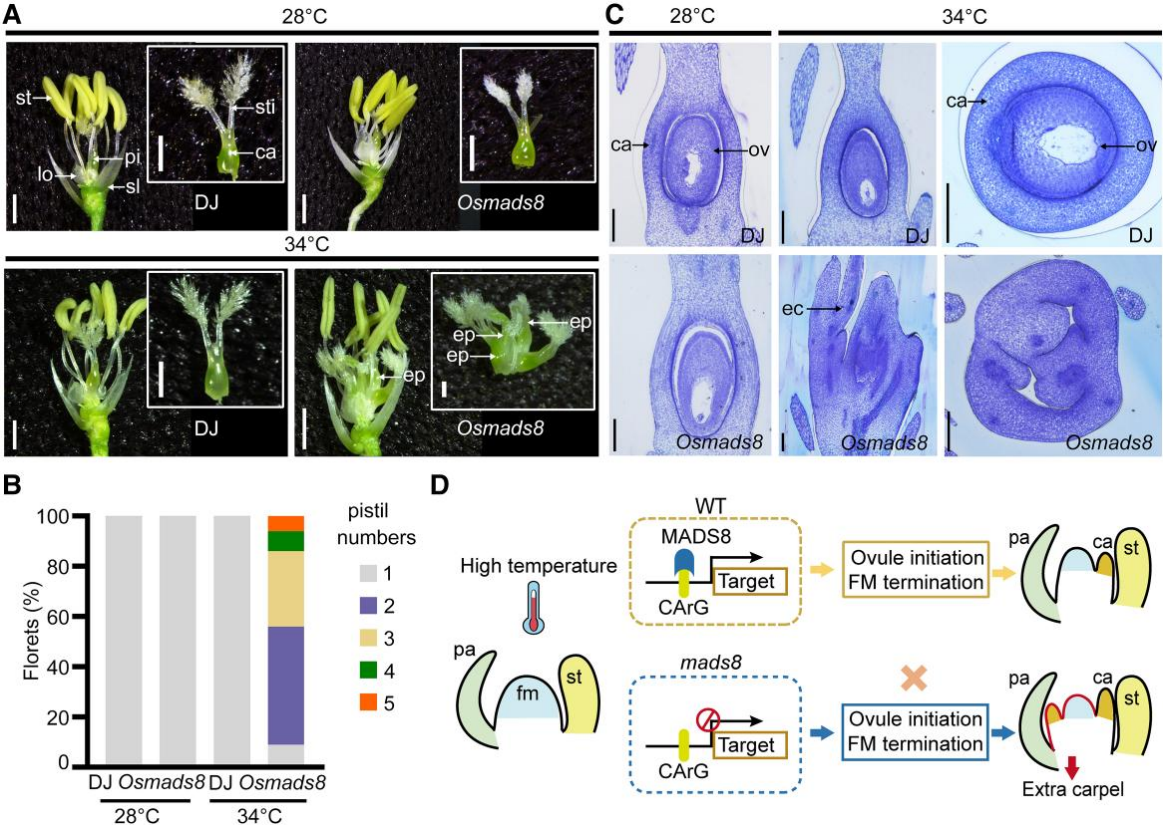
MADS8 function in temperature-dependent pistil regulation is conserved between barley and rice. **A)** Pistil phenotypes of DJ (WT) and *Osmads8* mutants grown at control (28 °C average) and high (34 °C average) temperatures. ca, carpel; ep, extra pistil; lo, lodicule; pi, pistil; sl, sterile lemma; st, stamen; sti, stigma. Scale bars, 900 *μ*m. **B)** Pistil number in *Osmads8* florets grown at different temperatures. **C)** Semi-thin sections of stage 12 DJ and *Osmads8* pistils at 28 or 34 °C. ca, carpel; ec, extra carpel; ov, ovule. Scale bars, 100 *μ*m. **D)** Proposed model of HvMADS8-mediated pistil development regulation at high temperatures. In WT flowers, MADS8 binds target genes to induce ovule initiation and FM termination at the appropriate developmental stage under a specific high-temperature signaling pathway. In the absence of MADS8, continued FM development triggers the repeated formation of extra carpels in the innermost whorl. fm, floral meristem; pa, palea.

Previous studies have shown no obvious alterations in either vegetative or reproductive organ phenotypes in *OsMADS8* single knockdown lines (Cui et al. 2010). To assess the function of OsMADS8 at different temperatures, we created a rice *Osmads8* mutant that contains a 2-bp insertion within the first exon in the Dongjin (DJ) background (Supplemental Fig. S1A). Consistent with previous reports from RNA interference (RNAi) lines, the loss-of-function *Osmads8* mutant obtained here displayed no significant floral organ defects at control temperatures (28 °C; Fig. 7A). However, we determined that *Osmads8* plants display a lower seed setting rate and shrunken grain morphologies compared to WT at 28 °C (Supplemental Fig. S4, A to D), indicating a key role for OsMADS8 during postfertilization development. At higher temperatures (34 °C), the *Osmads8* mutant also produced multiple pistils with no noticeable changes in other flower organs, as in barley (Fig. 7, A and B). Histological analysis of preanthesis rice florets also revealed an ovule initiation defect in *Osmads8* mutant pistils at 34 °C, resulting in the failure of grain production at high temperatures (Fig. 7C; Supplemental Fig. S4E). Collectively, our data suggest a conserved function for MADS8 between rice and barley in maintaining the developmental stability of FM and pistil structures in a temperature-specific manner. As shown in our proposed model (Fig. 7D), downstream genes in flower development pathways are regulated by MADS8 to drive ovule initiation and FM determinacy in WT pistils at high temperatures. In *mads8* mutants, the lack of MADS8 leads to the iterative formation of carpels and failure to initiate ovule primordia at high temperatures (Fig. 7D).

## Discussion

### Barley SEP members stabilize reproductive development through changes in their DNA binding affinity at high ambient temperature

Several studies have identified MADS-box transcription factors as key components of a plant regulatory network that integrates environmental cues to affect responses at the cellular level but most are involved in flowering time control (Trevaskis et al. 2003; Sureshkumar et al. 2016; Whittaker and Dean 2017). Here, we report that *Hvmads8* exhibits temperature-dependent defects in female organs and FM during gynecium development (Figs. 1 and 2). Moreover, using the CUT&Tag approach, we revealed the genome-wide downstream binding targets of a MADS-box protein at high temperatures (Fig. 5, A to C). By integrating these results with RNA-seq data, we propose that at high temperatures, increased binding of HvMADS8 to the promoters of genes in flower and meristem development pathways contributes to pistil developmental stability (Figs. 5 and 7D).

Compared to our previous discovery that the *LOFSEP* member *HvMADS1* regulates barley inflorescence shape stability (Li et al. 2021), here we show that the *SEP3* member *MADS8* modulates temperature-responsive female organ development. Unlike the branch-like inflorescence of *Hvmads1* under high temperatures, the defects of *Hvmads8* mutants are restricted to the innermost floral whorl, i.e. meristem indeterminacy and extra pistil-like structures at high temperatures (Figs. 1 and 2). Similarly, HvMADS8 and HvMADS1 have stable transcript and protein levels at different temperatures (Fig. 3, B and F); they fulfill their thermal response functions through temperature-sensitive binding to the promoters of their downstream target genes (Fig. 5). These findings on the barley SEP family extend the known function of floral homeotic proteins in linking them to thermal responsiveness during reproductive development. Further investigations on higher-order mutants of *SEP* family members, and other floral homeotic genes at different temperatures, may provide more information about the mechanistic framework underlying reproductive organ thermal response. The single mutant *Hvmads7* displays no noticeable differences in pistil development at either control or high temperatures (Supplemental Fig. S5, A and B). However, double knockout mutants for the 2 *SEP3*-like genes *HvMADS7* and *OsMADS8* display extra lemma and palea in florets, and lack inner floral organs at the control temperature (15 °C), suggesting a conserved redundant function among E-class genes of barley, rice, and Arabidopsis in controlling the inner floral organ identities at normal growth temperature conditions (Supplemental Fig. S5, A and C) (Pelaz et al. 2000, 2001; Wu et al. 2018). The contribution of HvMADS7 and other floral homeotic proteins in temperature-responsive floral development remains to be explored.

### MADS8 directs ovule initiation and FM determinacy at high temperatures

The pattern of FM termination is diverse among angiosperm species. In Arabidopsis, stem cell maintenance ceases during carpel primordium initiation, and then ovules differentiate from the placenta inside carpels (Cucinotta et al. 2014). The carpel identity gene *AG* can, directly and indirectly, repress the key FM regulator *WUSCHEL* to arrest floral stem cell activity for proper carpel development (Liu et al. 2011; Sun et al. 2019). In rice, after stamen and carpel differentiation, the FM is exhausted and ovules are finally produced from the terminating FM (Yamaki et al. 2011). Loss of function of the ovule identity gene, *OsMADS13*, leads to FM termination defects (Dreni et al. 2007). Thus, the central region of the FM is consumed by the carpel or ovule primordium in Arabidopsis and in rice, respectively, and carpel identity genes in the innermost whorl play indispensable roles in FM determinacy control. Barley may share a similar FM termination mechanism as rice. Unlike the extra carpels produced in rice *fon* mutants and wheat tri-pistil mutants that can produce multiple grains within a single floret (Jiang et al. 2005; Chu et al. 2006; Moon et al. 2006; Suzaki et al. 2006; Liu et al. 2022), extra carpels in *Hvmads8* florets form at the expense of ovules at high temperatures (Fig. 2A), indicating a close link between ovule initiation and FM determinacy during the floral developmental program. Based on similar defects observed in *Osmads13* mutant lines, we speculate that barley D-class genes also function in the ovule and FM regulatory pathway and contribute to the *Hvmads8* phenotype, where their expression is downregulated at high temperatures (Fig. 2, B and C; Supplemental Fig. S2, J and K). We confirmed the direct regulation of *HvMADS13* and *HvMADS21* by HvMADS8 (Fig. 6), although we failed to identify binding sites in the promoters of these 2 genes by CUT&Tag analyses at a relatively strict threshold (*P* ≤ 0.01). One possible explanation is that the strong direct binding of HvMADS8 to these 2 D-lineage genes occurs at later stages, as there is a stage difference between the samples used for CUT&Tag (W4.5 inflorescence) and ChIP-qPCR (W5.5 to W6.5 inflorescence). Nevertheless, single and double mutants of barley D-class genes are needed to uncover their precise genetic functions in ovule initiation and FM development.

### The potential roles of protein interaction networks in HvMADS8 function

MADS-box proteins interact with each other to determine flower formation and floral organ specification (Theissen et al. 2016; Abraham-Juárez et al. 2020). In Arabidopsis, SEP3 functions as a central hub to drive protein–protein interaction with other MADS-box transcription factors (Immink et al. 2009). Furthermore, the heterotetramer formed by SEP3 and the C-class protein AG is necessary for meristem determinacy through the activation of *KNUCKLES* and *CRABSCLAW* (Hugouvieux et al. 2019). Additionally, the variant SEP3^Δtet^ produced from a splice variant in Arabidopsis, lacking heterotetramerization capacity, was able to restore the first 3 whorls in the *sep1 sep2 sep3* triple mutant background but failed to rescue the unfused carpel and indeterminate flower-within-a-flower phenotype of this triple mutant. Thus, tetramerization of SEP3 with other floral homoeotic proteins, probably AG, may be required to activate a subset of target genes to terminate the FM (Hugouvieux et al. 2019).

Our work further reveals that HvMADS8 can interact with other C-, D-, E-class, and AGL6 subfamily homeotic proteins, so it is possible that protein–protein interactions also contribute to HvMADS8-regulated gene expression at high temperatures (Supplemental Fig. S3). Although we observed limited temperature-sensitive differences in protein–protein interactions in yeast, other assays, such as bimolecular fluorescence complementation (BiFC), or truncated HvMADS8 variants that affect protein–protein interactions, may be needed to provide clarity regarding interaction networks between HvMADS8 and floral homeotic proteins at various temperatures. Besides, sequence analyses comparing the barley E-class proteins to the B- and the C-class proteins, which have no temperature-responsive regulation to CArG-box elements (Muiño et al. 2014; Li et al. 2021; Selva et al. 2023), revealed a very conserved DNA-binding MADS domain from all families, while the C-terminal domain, involved in transcriptional activity (Muiño et al. 2014; Callens et al. 2018), showed a huge diversity among these temperature-dependent or temperature-independent members (Supplemental Fig. S6).

### Conserved MADS8 function in barley and rice during carpel development

Studies in key model plants have shown that the formation of various floral organs is coordinated by the actions and interactions among MADS-box family proteins (Gramzow and Theissen 2010; Theissen et al. 2016). Interestingly, many of these factors show conserved function in flower development among species (Ciaffi et al. 2011; Murai 2013; Wu et al. 2018; Kuijer et al. 2021). In this study, we analyzed loss-of-function *mads8* mutants in barley and rice, with both mutants displaying increased carpels and loss of ovules specifically at high temperatures (Figs. 1 and 7). Although the normal growth temperature differs between barley and rice (15 to 20 and 28 °C, respectively) due to their distinct domestication trajectories (Fuller et al. 2009; Pankin and von Korff 2017), MADS8 has a conserved temperature-dependent function (Figs. 1 and 7). The unaffected levels of *HvMADS1* and *HvMADS8* mRNA and HvMADS1 and HvMADS8 protein (Fig. 3, B and F) (Li et al. 2021) under high temperature led to our speculation that these MADS-proteins may undergo conformational changes to enhance their DNA binding affinity and facilitate global transcriptional adjustments to high temperatures during reproduction, which remains to be investigated.

Analyses of single nucleotide polymorphisms (SNPs) using the rice SNP-Seek Database derived from 3,000 rice genomes revealed no SNP within the coding regions of *OsMADS8* (Wang et al. 2018); analyses of 267 barley genomes also revealed strong conservation of the MADS8 sequence among different accessions (Russell et al. 2016) (Supplemental Table S1). Overall, our study indicates that MADS8 appears to have a conserved function in determining pistil development and terminating FM in a temperature-dependent manner in at least 2 grass species. In Arabidopsis, SEP3 also plays a similar role as MADS8 in grasses in terms of FM determination and carpel regulation. Floral organs are converted to sepaloid-like structures in Arabidopsis *sep1 sep2 sep3* triple mutants with a lack of FM determinacy, resulting in carpels being replaced by continuous generation of sepals (Pelaz et al. 2000).

It will be interesting to examine whether more conserved regulators in flower developmental pathways are shared between crop species. Knowledge of the mechanisms governing floral organ development in restrictive temperatures, and whether they are conserved across cereal crops, might offer crucial insights that can be used in the context of global warming to engineer plants with improved developmental stability.

## Materials and methods

### Plant materials, CRISPR design, and plant transformation

The barley (*H. vulgare*) cultivars used in this study were ‘GP’ (United Kingdom) and ‘WI’ (Australia). The monocot CRISPR/Cas9 system (Ma et al. 2015) was used to obtain barley edited lines. Two single guide RNAs (sgRNAs) were designed to target the first exon of *HvMADS8* and *HvMADS7* within the sequence encoding the MADS domain (http://skl.scau.edu.cn/targetdesign/) (Supplemental Figs. S1A and S5A). Target specificity was confirmed by BLAST of the barley genome (http://plants.ensembl.org/Hordeum_vulgare/Tools/Blast). Target sites sequenced in GP and WI all showed 100% identity to the ‘Morex’ reference genome. The sgRNA expression cassettes driven by the promoters *OsU6a* and *OsU6b* were cloned into the PYLCRISPR-Cas9Pubi-H binary vector (Li et al. 2021). *Agrobacterium* (*Agrobacterium tumefaciens*)-mediated transformation (strain AGL1) of immature barley embryos was performed for both cultivars (Harwood 2014). Independent T_0_ plants were genotyped using a Phire Plant Direct PCR Kit (Thermo Fisher Scientific), and the amplified target sites were Sanger sequenced (AGRF, Australia).

The rice (*O. sativa*) cultivar ‘DJ’ (ssp. *japonica*) was used to generate the *Osmads8* CRISPR/Cas9 knockout mutant as previously described (Xie et al. 2015). Genotyping of *Osmads8* lines was performed by PCR amplification from genomic DNA extracted from the T_1_ plants, followed by Sanger sequencing (Personalbio, Shanghai).

To investigate HvMADS8 protein accumulation and function, *proHvMADS8:HvMADS8*-*eGFP* transgenic lines were created by *Agrobacterium*-mediated transformation as described above. The 2,870 bp *HvMADS8* promoter and full-length cDNA were cloned in-frame and upstream of the *eGFP* sequence into pCAMBIA1301, using the In-Fusion (Takara) cloning technology with *Kpn*I and *Nco*I sites. The resulting construct was transformed into the GP WT barley background. The primers are listed in Supplemental Data Set 5.

### Plant growth conditions and temperature treatments

All barley plants were germinated on cocopeat soil at control temperatures (15 °C day/10 °C night), with 50% humidity and a 16-h light/8-h dark photoperiod in growth chambers with a light intensity of ∼190 *μ*mol m^−2^ s^−1^ (The Plant Accelerator, University of Adelaide; Conviron GR48, Shanghai Jiao Tong University). For high-temperature treatments, plants were grown at the control temperature until Waddington stage W1 and then were moved to higher temperatures (20, 25, or 28 °C) for phenotyping or sample harvesting. Night (dark) temperatures were 5 °C below the day (light) temperature for all temperature treatments.

Rice plants were grown at an average temperature of 28 °C with 14-h light/10-h dark photoperiod and 75% relative humidity in a growth chamber (Conviron GR48) as control conditions. For high-temperature treatments, plants were first grown in control conditions; at the onset of reproductive development, plants were moved to a growth chamber with an average temperature of 34 °C.

### Morphological analysis and microscopy observation

Fresh pistils from WT and mutant florets were photographed using a stereomicroscope equipped with a digital camera (Leica, MZ FLIII and S8AP0). Immature inflorescence and pistil tissues from plants grown at different temperatures were collected at various stages and prepared for SEM (Philips, XL30 FEG) as previously described (Wang et al. 2021). For cytological analysis, pistils at W9.5 were collected and fixed in formalin–aceto–alcohol (FAA) solution (50% [v/v] ethanol, 10% [v/v] formalin, and 5% [v/v] acetic acid) overnight and then dehydrated in a graded ethanol series. Samples were embedded in Technovit 7100 resin (Hereaus Kulzer). Transverse 2-μm sections were cut with an Ultratome III ultramicrotome (LKB) and then stained with 1% (w/v) toluidine blue and photographed using an optical microscope (Ni-E, Nikon and E600, Nikon). All confocal fluorescent images for *proHvMADS8:HvMADS8*-*eGFP* lines were captured using an Olympus IXplore SpinSR microscope from inflorescences and florets at various stages of development (excitation 488 nm; emission 500 to 530 nm).

### RNA extraction and RT-qPCR analysis

Total RNA was extracted from 3 biological replicates using TRIzol reagent (Invitrogen) from barley and rice samples. Samples included different stages of barley inflorescence (W2.0 to 7.5) and pistil (W8.0 to 10.5) development; the lemma, palea, awn, stem, and leaves from W9.5 florets and plants; and root samples from young seedlings. The young inflorescences in *Hvmads8* mutants were collected based on the awn and stamen primordia stages as in WT plants, and the various stages of pistils dissected from *Hvmads8* were determined according to the lemma and anther morphological changes in WT florets.

Two micrograms of total RNA was used for first-strand cDNA synthesis using a FastKing RT kit with gDNase (Tiangen Biotech). qPCR of diluted cDNA was performed using a 384-well QuantStudio Flex 6 (Thermo Fisher Scientific) instrument with a QuantiNova SYBR Green PCR Kit (Qiagen). The *HvActin7* gene was used as internal control as described previously (Li et al. 2021). All primers are listed in Supplemental Data Set 5.

### Immunoblotting of HvMADS8

Nuclear protein was extracted from W5.5 to 6.5 inflorescences collected from 2 independent *proHvMADS8:HvMADS8*-*eGFP* lines using nuclear protein extraction buffers as described previously for ChIP assays (Bowler et al. 2004). The concentrations of isolated protein samples were measured using a Bradford Protein Assay Kit (Sangon Biotech). Equal amounts of protein from each sample were separated on 12.5% (w/v) SDS-PAGE using a PAGE Gel Fast Preparation Kit (EpiZyme Biotechnology) and transferred onto PVDF membrane (Bio-Rad). The membrane was incubated with primary antibodies against GFP (1:1,000 dilution; Proteintech 66002-1-Ig) or tubulin (1:2,000 dilution; Beyotime AT819) at 4 °C overnight and with secondary antibodies conjugated to horseradish peroxidase (1:5,000 dilution; Abmart M21001) for 1 h at room temperature. Immunoreactive bands were visualized with a Chemidoc MP Imaging System (Bio-Rad) using SuperSignal West Pico chemiluminescent substrate (Pierce).

### In situ hybridization

Barley inflorescences were fixed immediately and embedded for in situ hybridization as previously described (Wu et al. 2018). Fragments of *HvDL* (314 bp), *HvMADS13* (350 bp), *HvOSH1* (327 bp), and *HvMADS8* (302 bp) were amplified from WT cDNA as probe templates with specific primers fused to the T7 promoter (Supplemental Data Set 5). Digoxigenin-labeled antisense and sense probes were transcribed using a DIG RNA Labeling Kit (SP6/T7; Roche) according to the manufacturer's instructions. An InsituPro VSi robot (Intavis) was used to perform the in situ hybridizations automatically following a standard protocol, as described before (Javelle et al. 2011; Li et al. 2021). Images were photographed by an optical microscope (Ni-E, Nikon).

### RNA sequencing and data analysis

Inflorescence (W4.0 and W4.5) and pistil (W8.0) samples were collected from WT (GP) and *Hvmads8* plants grown at control and high temperatures. Total RNA from 3 biological replicates was extracted using TRIzol reagent (Invitrogen). RNA-seq library preparation and sequencing were performed on an Illumina HiSeq4000 platform by Novogene (China). The quality of raw reads was examined and reads were trimmed; clean reads were mapped to the barley reference genome (Barley cv. Morex IBSC 2 V1 2016, https://galaxy-web.ipk-gatersleben.de/libraries/folders/Ff2db41e1fa331b3e) as described previously (Mascher et al. 2017; Li et al. 2021). The count data were calculated using HTSeq. Transcripts per million (TPM) values were normalized using a Python script. Further analysis was based on 39,734 high-confidence genes. The scatterplots for correlation analyses were generated using log_2_ fold change of gene expression at 28 °C compared to 15 °C at each stage, and a custom R script was used to calculate linear relationships (Jung et al. 2016). The R package DESeq2 (version 1.32.0) was used to identify DEGs that correlated with genotype (WT relative to *Hvmads8*) or temperature (28 °C compared to 15 °C). The false discovery rate (FDR)-adjusted *P*-values were computed by the Benjamini–Hochberg model. Genes were considered to be differentially expressed if both the |log_2_ fold change| > 0.585 and the *P*-adjust < 0.05 (Supplemental Data Set 1). DEGs were annotated using the BioMart database of Ensembl Plants. Information on homologs in Arabidopsis (TAIR10_peptide; http://www.arabidopsis.org/) and rice (MSU7_peptide; http://rice.plantbiology.Msu.edu/) was retrieved from the listed websites. Volcano plots were created using log_2_ fold change of DEGs at 15 and 28 °C with the R package EnhancedVolcano (Blighe et al. 2019).

GO analysis was performed with the R package (topGO) for DEGs according to Arabidopsis annotations (Supplemental Data Set 2). GO terms were considered to be significantly enriched with a corrected FDR < 0.05. Treemaps were used to visualize GO term results (Li et al. 2021). Genes related to meristem regulation, flower development, and temperature response were selected for heatmap analysis using MeV software (mev.tm4.org; Supplemental Data Set 3).

### ChIP-qPCR

Approximately 1 g of W5.5 to 6.5 inflorescence was collected from one line of *proHvMADS8:HvMADS8*-*eGFP* plants grown at control or high temperatures. Samples were crosslinked with 1% (v/v) formaldehyde in extraction buffer (0.4 m sucrose, 10 mm Tris-HCl, pH 8.0, 5 mm β-mercaptoethanol, and 0.1 mm PMSF), and the ChIP assay was performed as described previously (Zhu et al. 2022). GFP-Trap and Binding Control Magnetic Agarose (ChromoTek) were used for chromatin precipitation at the same time. Crosslinks were reversed with 0.2 m sodium chloride and overnight incubation at 65 °C. The extracted DNA was diluted 1:4, and the abundance of the promoter fragments from *HvMADS13* and *HvMADS21* was quantified by qPCR using a LightCycler 96 Real-Time PCR system (Roche) with a SYBR Green Premix *Pro Taq* HS qPCR Kit (Accurate Biology). The *HvActin7* gene was used as a negative control. All primers are listed in Supplemental Data Set 5.

### Dual-luciferase assays

The dual-luciferase (LUC) assay was performed in *N. benthamiana* leaves, from plants grown and treated as described before (Li et al. 2021). Full-length *HvMADS8* and *HvMADS3* cDNA was cloned into the pGreenII-0000 vector containing the cauliflower mosaic virus (CaMV) 35S promoter for effector plasmid construction. For reporter constructs, the promoters of *HvMADS13* and *HvMADS21* from GP were cloned upstream of the firefly luciferase (*LUC*) reporter gene into the pGreenII-0800-LUC vector, which also harbors the *Renilla* luciferase (*REN*) gene driven by the CaMV 35S promoter. The *proHvMADS13MU:LUC* construct contains a mutation in the CArG-box sequence in the P1 binding motif, changing the original CArG-box sequence from CCAATAATAG to ATGTGAATAG. The *35S:HvMADS1*, *proCArG*, and *proCArG-mu* effector and reporter constructs were used as described previously (Li et al. 2021). Primers are listed in Supplemental Data Set 5.

Effectors and reporters were individually transformed into *Agrobacterium* strain GV3101 containing the helper plasmid pSoup-P19, which encodes a repressor of cosuppression. *Agrobacterium* colonies harboring each effector construct were then coinfiltrated into *N. benthamiana* leaves with *Agrobacterium* colonies carrying the reporter construct. The empty effector vector pGreenII-0000 was used as negative control. *N. benthamiana* plants were grown in the dark for around 48 h at relatively low temperature (20 °C, no phenotypes shown for *Hvmads8*) or high (28 °C) temperatures before sample collection. LUC and REN activities were measured with a Dual-Luciferase reporter kit (Promega) according to the manufacturer's instructions using a GloMax-96 Microplate Luminometer (Promega). Five biological replicates were used for each effector and reporter combination.

### EMSAs

The full-length *HvMADS8* cDNA was amplified from inflorescence cDNA and cloned into the pGADT7 vector (Clontech) for in vitro translation using a TNT T7/SP6 Coupled Wheat Germ Extract System (Promega). The CArG-box and its flanking sequence in the promoter region of *HvMADS13* and *HvMADS21* were synthesized as FAM-labeled primers at Generay Biotech (China). 10× unlabeled probes were used as competitor. The probe preparation and assays were performed as described previously (Zhu et al. 2022). Primers are listed in Supplemental Data Set 5.

### CUT&Tag assay and data processing

The CUT&Tag assay was performed as previously described (Li et al. 2022), with minor modifications. Briefly, antibodies specific to GFP (Abcam, ab290), H3K4me3 (Abcam, ab8580; positive control), and IgG (Sigma, 12-370; negative control) were used as primary antibodies at 1:50 dilutions. Guinea pig antirabbit IgG (Abnova, PAB9407) was used as the secondary antibody at 1:100 dilution. Sequencing was performed on a NovaSeq instrument as 150-bp paired-end reads by Personalbio (China). All sequencing reads were cleaned with NGmerge to move adapters before mapping the clean reads to the barley reference genome (Barley cv. Morex IBSC 2 V1 2016, https://galaxy-web.ipk-gatersleben.de/libraries/folders/Ff2db41e1fa331b3e) using Bowtie2 with *--end-to-end --very-sensitive --no-mixed -I 10 -X 700* as parameters (Mascher et al. 2017). The aligned BAM files were sorted for further analysis using Samtools; the peaks were called using Genrich with the threshold *P*-value set to 0.01. Peaks were merged using Bedtools between groups (Quinlan and Hall 2010). The ChIPseeker package was used to analyze the HvMADS8 binding positions (Yu et al. 2015). Genes identified in both biological replicates with peaks located within ±3 kb of the gene translation start site (TSS) were deemed potential targets (Supplemental Data Set 4). Bound peaks were visualized using Integrative Genomics Viewer (version 2.11.2) (Robinson et al. 2011).

### Yeast 2-hybrid assays

The full-length cDNAs of *HvMADS3*, *HvMADS58*, *HvMADS13*, *HvMADS21*, *HvMADS1*, *HvMADS5*, *HvMADS34*, *HvMADS7*, *HvMADS8*, and *HvMADS6* were individually amplified with gene-specific primers from inflorescence cDNA (Supplemental Data Set 5). The amplified *HvMADS8* sequence was then cloned into the prey activation domain (Ad) vector pGADT7; the other sequences were cloned into the bait DNA-binding domain (BD) vector pGBKT7 (Clontech), using the In-Fusion (Takara) cloning technology at the *Eco*RI and *Bam*HI sites. These constructs were transformed using one-step buffer (0.1 m dithiothreitol; 0.3 m lithium acetate and 40% [w/v] polyethylene glycol 4000) followed by a 30-min incubation at 45 °C into yeast (*Saccharomyces cerevisiae*) strain AH109 (BD Biosciences, USA), and the yeast 2-hybrid assay was performed according to the MATCHMAKER GAL4 Two-Hybrid System 3 & Libraries User Manual (Clontech). Yeast transformants were selected on SD medium lacking Leu and Trp (SD/–Leu/–Trp, SD–2). Protein interactions were tested, with no detectable self-activation for each of the single constructs on SD medium with high stringency (SD–Ade/–His/–Leu/–Trp, SD–4). To compare interactions at different temperatures, transformants grown on SD–2 medium were used as a reference. Transformants containing empty plasmids pGADT7 (prey) and pGBKT7 (bait) were used as negative controls.

### Amino acid sequence alignment

The full-length amino acid sequence of HvMADS2, HvMADS4, HvMADS16, HvMADS3, HvMADS58, HvMADS1, HvMADS5, HvMADS34, HvMADS7, and HvMADS8 were imported into SnapGene (Version 4.3.7), and the amino acid alignment was conducted using MUSCLE alignment. The HvMADS8 protein sequence was used as the reference.

### Variation of *HvMADS8*

The exome-sequencing data derived from 267 barley accessions were analyzed (https://www.ebi.ac.uk/ena/data/view/PRJEB8044) (Russell et al. 2016). Morex_contig_350004, Morex_contig_6154, and Morex_contig_2548971, which contain the *HvMADS8* gene sequence, were used for SNP identification. SNP calling was performed by visual inspection of the sequences (Supplemental Table S1).

### Quantification and statistical analysis

Statistical analysis was performed with Excel (Microsoft) or GraphPad Prism 8.0.2. For the comparison of 2 data groups, 2-way ANOVA was used to evaluate significance, with values given as means ± Sd. At least 3 biological repeats are displayed. All analyses are summarized in Supplemental Data Set 6.

### Accession numbers

The sequences for all barley genes used in this study are available in the Phytozome database (https://phytozome-next.jgi.doe.gov/). Rice gene sequences are available in the Rice Genome Annotation Project database (http://rice.uga.edu/analyses_search_locus.shtml). The accession numbers of the key genes mentioned are as follows: *HvMADS8* (HORVU5Hr1G076400), *HvDL* (HORVU4Hr1G067780), *HvMADS13* (HORVU1Hr1G023620), *HvOSH1* (HORVU4Hr1G009730), *HvMADS21* (HORVU1Hr1G064150), *HvMADS1* (HORVU4Hr1G067680), and *OsMADS8* (LOC_Os09g32948). The accession numbers of genes used in RT-qPCR and yeast 2-hybrid assay are listed in Supplemental Data Set 5. The raw data files for the CUT&Tag analysis reported in this paper have been deposited in the SRA database (accession no. PRJNA1005699).

## Supplementary Material

koad246_Supplementary_Data

## Data Availability

The authors declare that all data supporting the finding of this study are available within the manuscript and its supplementary information files or are available from the corresponding author upon request.

## Supplemental data

The following materials are available in the online version of this article.

**Supplemental Figure S1**. Genotypes and phenotypes of *Hvmads8* mutants in the GP and WI WT backgrounds.

**Supplemental Figure S2**. Mutation of *HvMADS8* alters expression of floral- and meristem-related genes at high temperature.

**Supplemental Figure S3**. Interaction of HvMADS8 with barley homologs of homeotic regulatory proteins at different temperatures.

**Supplemental Figure S4**. Seed setting of *Osmads8* at different temperatures.

**Supplemental Figure S5**. Genotypes and phenotypes of *Hvmads7* single and *Hvmads7 Hvmads8* double mutants in the GP background.

**Supplemental Figure S6**. Amino acid sequence alignment of barley E-class family proteins with B- and C-class members.

**Supplemental Table S1**. Variation of *HvMADS8* coding sequences.

**Supplemental Data Set 1**. DEGs of *Hvmads8* in terms of different temperatures or genotypes.

**Supplemental Data Set 2**. GO analysis of DEGs in WT and *Hvmads8* at 15 and 28 °C.

**Supplemental Data Set 3**. Curated list of genes for heatmaps in RNA-seq data.

**Supplemental Data Set 4**. Target genes of HvMADS8 at 15 and 28 °C from CUT&Tag.

**Supplemental Data Set 5**. Primers used in this study.

**Supplemental Data Set 6**. Summary of statistical analyses.

## Dive Curated Terms

The following phenotypic, genotypic, and functional terms are of significance to the work described in this paper:

SEP3 Gramene: AT1G24260
SEP3 Araport: AT1G24260

KAN2 Gramene: AT1G32240
KAN2 Araport: AT1G32240

KNAT6 Gramene: AT1G23380
KNAT6 Araport: AT1G23380

SUP Gramene: AT3G23130
SUP Araport: AT3G23130

AP2 Gramene: AT4G36920
AP2 Araport: AT4G36920

PIF4 Gramene: AT2G43010
PIF4 Araport: AT2G43010

AGL6 Gramene: AT2G45650
AGL6 Araport: AT2G45650

OSH1 Gramene: Os03g0727000
OSH1 Araport: Os03g0727000

HvMADS8 Gramene: HORVU5Hr1G076400
HvMADS8 Araport: HORVU5Hr1G076400

OsMADS8 Gramene: Os09g0507200
OsMADS8 Araport: Os09g0507200

phyB Gramene: AT2G18790
phyB Araport: AT2G18790

OsMADS13 Gramene: Os12g0207000
OsMADS13 Araport: Os12g0207000

OsMADS21 Gramene: Os01g0886200
OsMADS21 Araport: Os01g0886200

OsMADS3 Gramene: Os01g0201700
OsMADS3 Araport: Os01g0201700

OsMADS58 Gramene: Os05g0203800
OsMADS58 Araport: Os05g0203800

OsMADS1 Gramene: Os03g0215400
OsMADS1 Araport: Os03g0215400

OsMADS5 Gramene: Os06g0162800
OsMADS5 Araport: Os06g0162800

OsMADS34 Gramene: Os03g0753100
OsMADS34 Araport: Os03g0753100

OsMADS7 Gramene: Os08g0531700
OsMADS7 Araport: Os08g0531700

HvMADS1 Gramene: HORVU4Hr1G067680
HvMADS1 Araport: HORVU4Hr1G067680

DL Gramene: Os03g0215200
DL Araport: Os03g0215200

HvDL Gramene: HORVU4Hr1G067780
HvDL Araport: HORVU4Hr1G067780

HvOSH1 Gramene: HORVU4Hr1G009730
HvOSH1 Araport: HORVU4Hr1G009730

HvMADS13 Gramene: HORVU1Hr1G023620
HvMADS13 Araport: HORVU1Hr1G023620

HvMADS21 Gramene: HORVU1Hr1G064150
HvMADS21 Araport: HORVU1Hr1G064150

HvMADS31 Gramene: HORVU2Hr1G098930
HvMADS31 Araport: HORVU2Hr1G098930

HvMADS29 Gramene: HORVU6Hr1G032220
HvMADS29 Araport: HORVU6Hr1G032220

HvMADS3 Gramene: HORVU3Hr1G026650
HvMADS3 Araport: HORVU3Hr1G026650

HvMADS58 Gramene: HORVU1Hr1G029220
HvMADS58 Araport: HORVU1Hr1G029220

HvMADS6 Gramene: HORVU6Hr1G066140
HvMADS6 Araport: HORVU6Hr1G066140
