# Supplementary material for: MADS8 is indispensable for female reproductive development at high ambient temperatures in cereal crops
Source: Plant Cell. 2023 Sep 21;36(1):65–84. doi: 10.1093/plcell/koad246 (PMC10734617; doi:10.1093/plcell/koad246)
Supplement: koad246_Supplementary_Data [file koad246_supplementary_data.zip › TPC2023RA00126R2_Supplemental_Data.pdf]

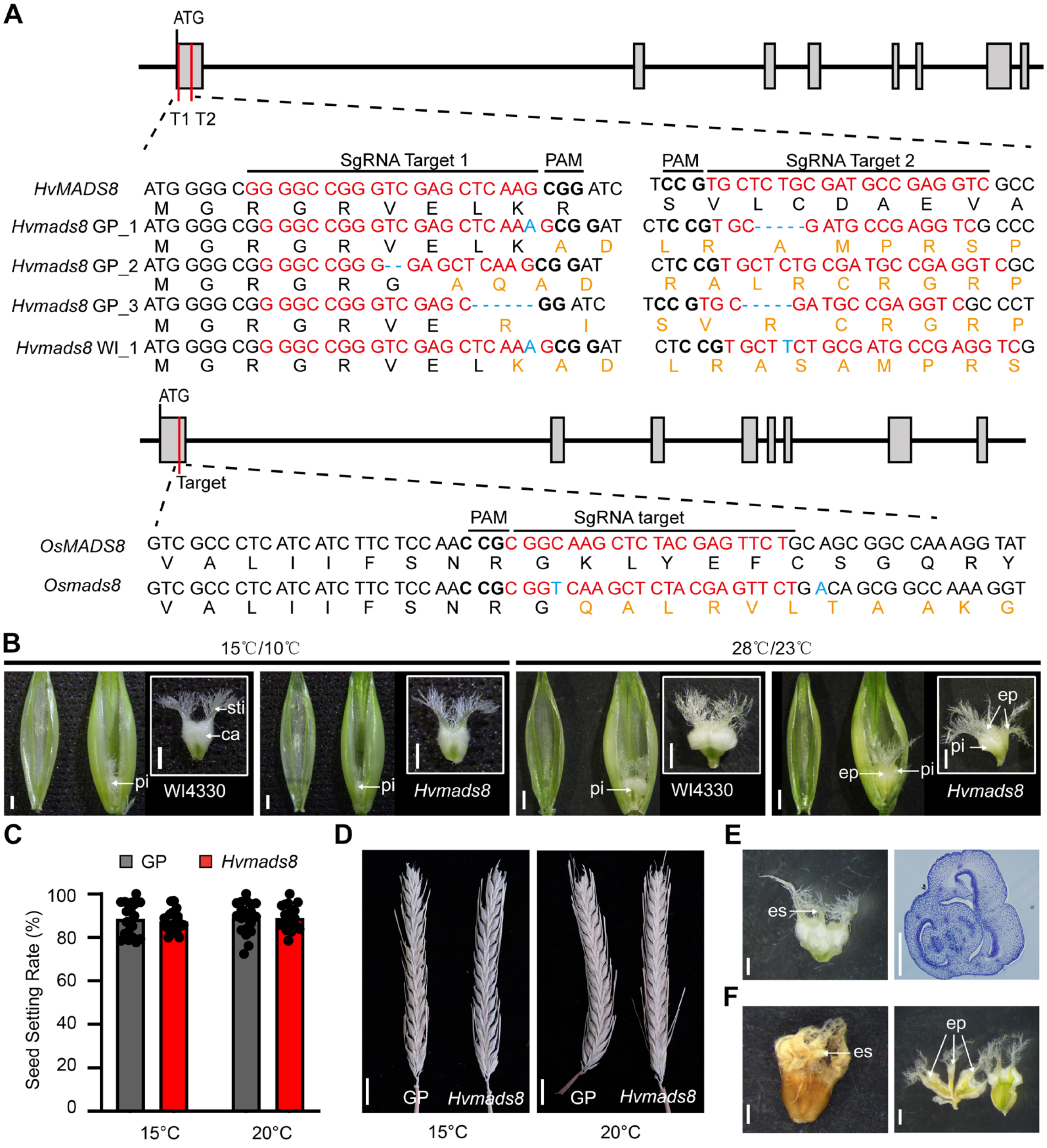

Supplemental Figure S1. Genotypes and phenotypes of *Hvmads8* mutants in the Golden Promise (GP) and WI4330 (WI) wild-type backgrounds. (Supports Figure 1)

(A) Diagram of the *HvMADS8* and *OsMADS8* loci (exons, gray boxes), showing positions and sequences of the sgRNA target sites. sgRNA target sequences (red) and protospacer adjacent motif (PAM, bold) are indicated. Blue indicates mutant nucleotide sequences (insertions and deletions/dashes), orange illustrates changed amino acids.

(B) Pistil phenotypes of wild-type (WT) and *Hvmads8* mutants in WI4330 grown under control (15°C day/10°C night) and high (28°C day/23°C night) temperature conditions. ca, carpel; ep, extra pistil;

pi, pistil; sti, stigma. Scale bars, 1 mm.

(**C**) Seed setting rate of GP and *Hvmads8* (GP) at 15°C and 20°C.

(**D**) Mature spikes of GP and *Hvmads8* (GP) at 15°C and 20°C.

(**E**) Pistil phenotype (left) and semi-thin section (horizontal; right) of the florets with one pistil at 28°C. es, extra stigma. Scale bars, 1mm.

(**F**) Mature pistils of *Hvmads8* (GP) with one pistil (left) and multiple pistils (right) at 28°C. Scale bars, 1mm.

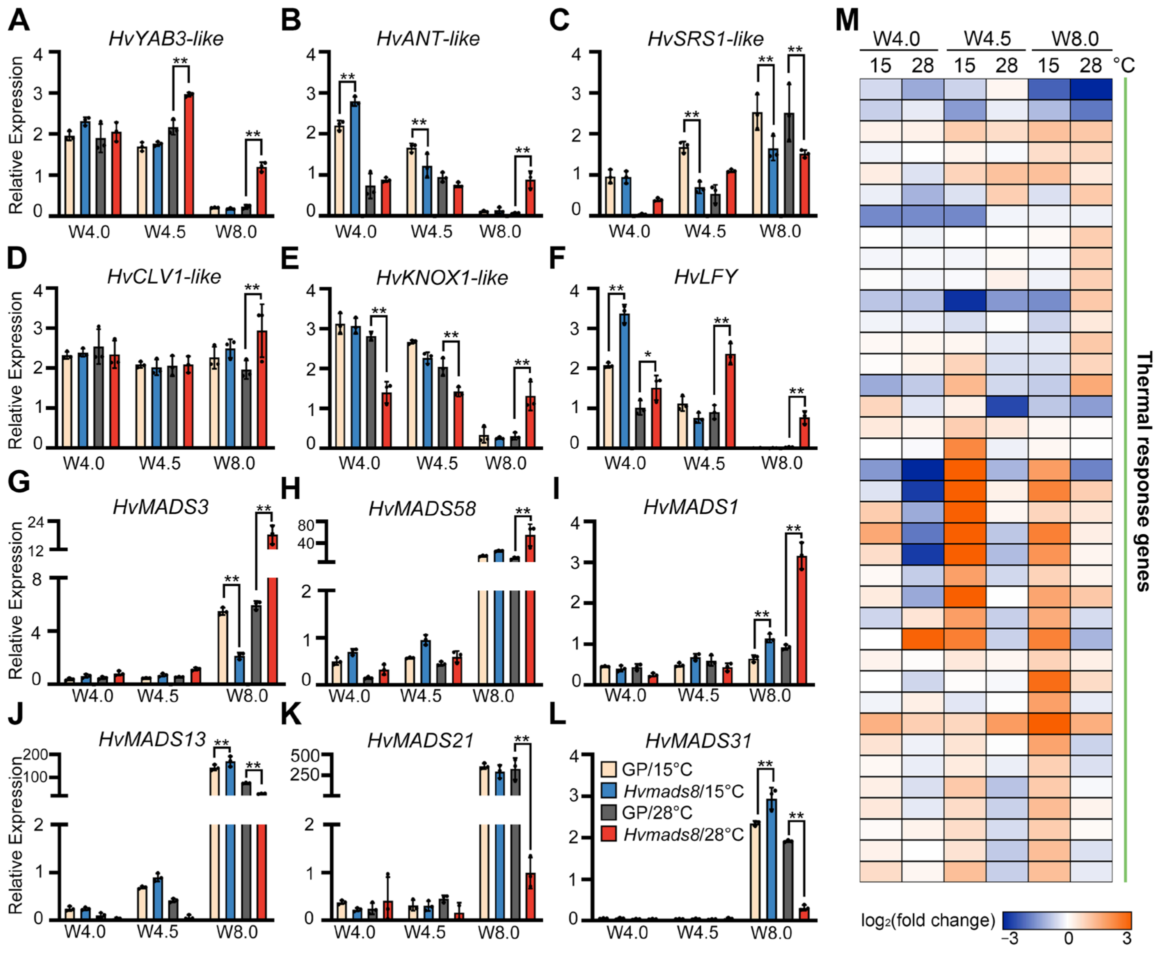

Supplemental Figure S2. Mutation of *HvMADS8* alters the expression of floral- and meristem-relevant genes at high temperature. (Supports Figure 4)

(A-L) Relative expression of key floral regulator genes in inflorescence and pistil samples used for RNA-seq from 15ºC and 28ºC. Values are means ± SD; *n* = 3 biological replicates. Asterisks indicate significant differences (two-way ANOVA test; **P*<0.05, ***P*<0.01).

(M) Heatmap representation of expression levels of thermally responsive DEGs, including genes involved in thermomorphogenesis and genes encoding heat shock proteins.

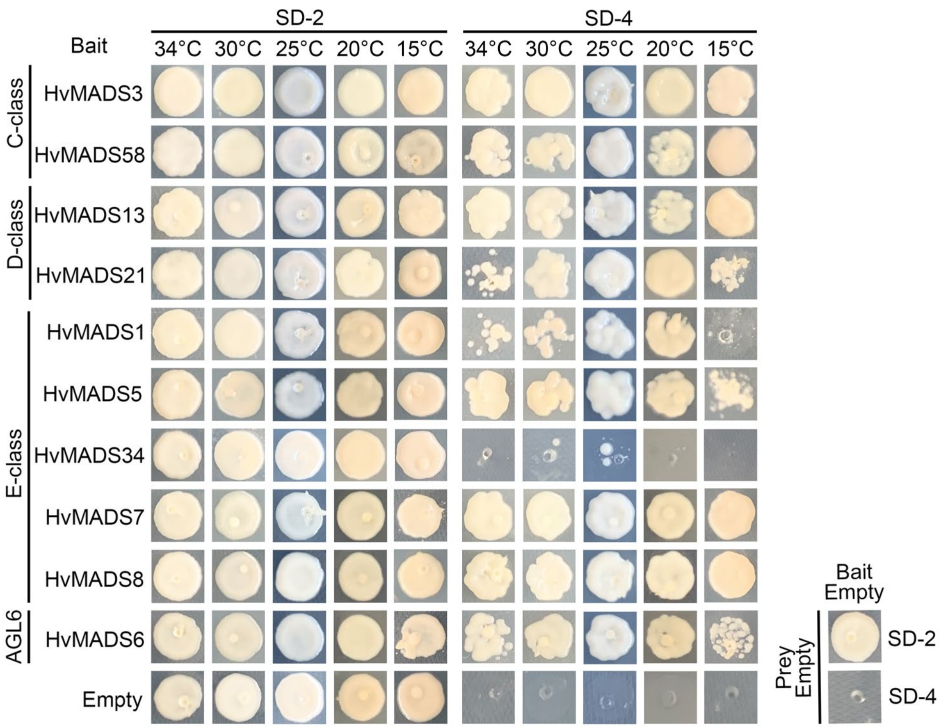

Supplemental Figure S3. Interaction of HvMADS8 with homeotic regulatory proteins at different temperatures. (Supports Figure 1)

Yeast two-hybrid assay showing the interaction of HvMADS8 (prey plasmid) with bait plasmids encoding barley class C (HvMADS3, HvMADS58); class D (HvMADS13, HvMADS21); class E (HvMADS1, HvMADS5, HvMADS34, HvMADS7, HvMADS8); and AGL6-like (HvMADS6) proteins. Transformants were grown on selective minimal synthetic defined (SD) medium at high stringency (SD–4; SD/–Ade/–His/–Leu/–Trp) at 34ºC, 30ºC, 25ºC, 20ºC and 15ºC to test interactions between MADS proteins; growth on SD–2 (SD–Leu–Trp) medium indicates presence of both bait and prey plasmids. Co-transformants with empty vectors pGADT7 (prey) and pGBKT7 (bait) were used as negative controls. Ade, adenine; His, histidine; Leu, leucine; Trp, tryptophan.

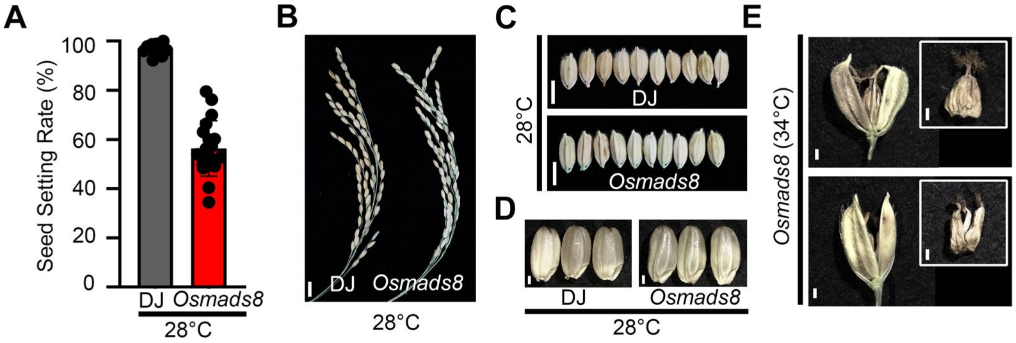

Supplemental Figure S4. Seed setting of *Osmads8* at different temperatures. (Supports Figure 7)

(**A**) Seed setting rate of DJ (Donjin) and *Osmads8* at 28°C.

(**B**) Mature panicles of DJ and *Osmads8* at 28°C. Scale bar, 1 cm.

(**C**) Seed phenotype of DJ and *Osmads8* at 28°C. Scale bars, 5 mm.

(**D**) Seed morphology with lemma and palea removed of DJ and *Osmads8* at 28°C. Scale bar, 1 mm.

(**E**) Mature florets and pistils of *Osmads8* with one pistil (top) and multiple pistils (bottom) at 34°C. Scale bars, 1 mm.

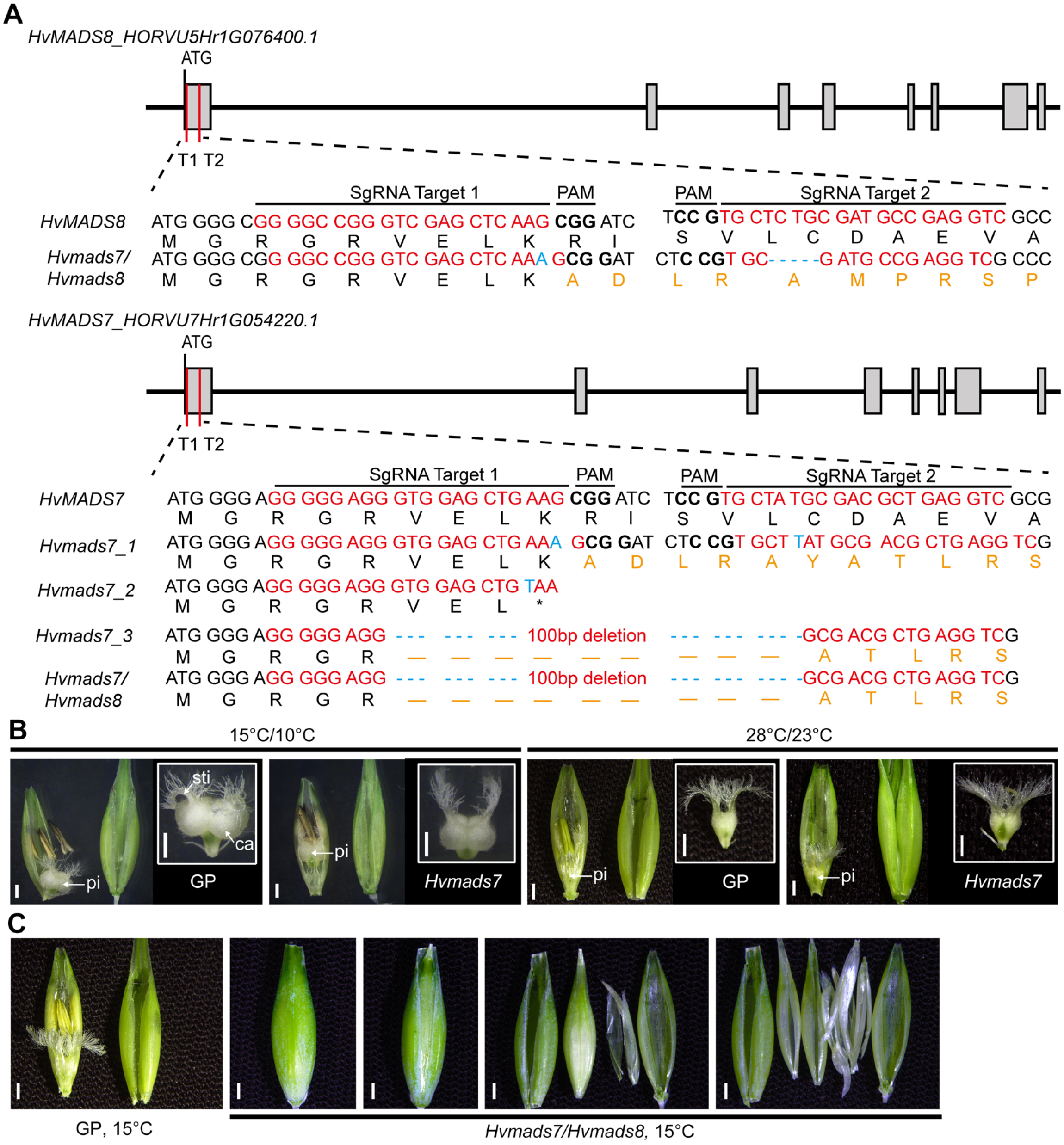

Supplemental Figure S5. Genotypes and phenotypes of *Hvmads7* single and *Hvmads7 Hvmads8* double mutants in the Golden Promise (GP) background at 15°C. (Supports Figure 1)

(**A**) Diagram of the *HvMADS8* and *HvMADS7* loci (exons, gray boxes) showing the position and sequence of the sgRNA target sites and the mutant sequence of the *Hvmads7* single and *Hvmads7 Hvmads8* double mutants. sgRNA target sequences (red) and protospacer adjacent motif (PAM, bold) are indicated. Blue indicates mutant nucleotide sequences (insertion and deletion/dashes), and orange illustrates changed amino acids.

(**B**) Floret phenotypes of WT and the *Hvmads7* single mutants in the GP background grown under control (15°C day/ 10°C night) and high (28°C day/23°C night) temperature conditions. ca, carpel; pi, pistil; sti, stigma. Scale bars, 1 mm.

(**C**) Floret phenotypes of WT and *Hvmads7 Hvmads8* double mutant in GP background grown under control (15°C day/ 10°C night) temperature conditions. Scale bars, 1 mm.

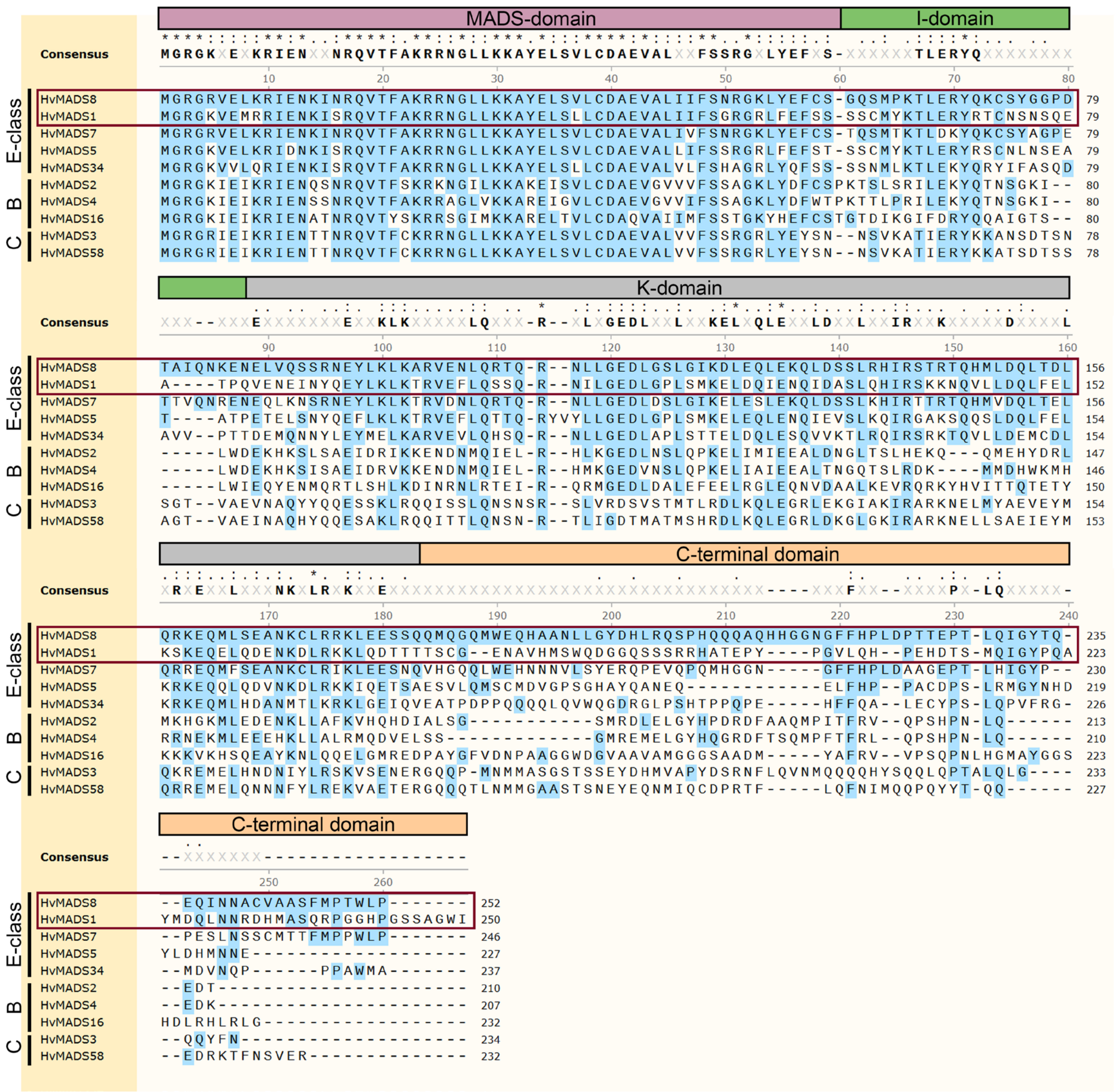

Supplemental Figure S6. Amino acid sequence alignment of barley E-class family proteins with B- and C-class members. (Supports Figure 6)

The HvMADS8 and HvMADS1, which display temperature-dependent functions, are marked in the brown box.

SNPs	Exon position	Domain	CDS position	Codons	Protein position	Amino acid	Frequency	Latitude range
1	1/8	MADS	108	UCC-UCU	36	Ser-Ser	1/267	31.7
2	2/8	I	229	GGG-UGG	77	Gly-Trp	4/267	24.6~32.1
3	2/8	I	237	GAU-GAA	79	Asp-Glu	1/267	36.1
4	3/8	K	279	AGU-AGC	93	Ser-Ser	1/267	30.1
5	3/8	K	285	AAC-AAU	95	Asn-Asn	4/267	39.0~42.0
6	3/8	K	288	GAG-GAA	96	Glu-Glu	1/267	30.1

Supplemental Table S1. Variation of *HvMADS8* coding sequences. (Supports Figure 7) Positions, codon changes, and latitude information of 6 SNPs identified in HvMADS8 exons across 267 barley varieties.
