# Supplementary material for: MADS8 is indispensable for female reproductive development at high ambient temperatures in cereal crops
Source: Plant Cell. 2023 Sep 21;36(1):65–84. doi: 10.1093/plcell/koad246 (PMC10734617; doi:10.1093/plcell/koad246)
Supplement: koad246_Supplementary_Data [file tpc23.00126Peer Review Report.pdf › tpc23.00126Peer Review Report.pdf]

MADS8 is indispensable for female reproductive development at high ambient temperatures in cereal crops

Chaoqun Shen, Yueya Zhang, Gang Li, Jin Shi, Duoxiang Wang, Wanwan Zhu, Xiujuan Yang, Ludovico Dreni, Matthew R. Tucker and Dabing Zhang

Corresponding author: Matthew R. Tucker, matthew.tucker@adelaide.edu.au

Review timeline:

**TPC2023-RA-00126**	Submission received:	Feb. 7, 2023
	1st Decision:	Mar. 26, 2023 *revision requested*
**TPC2023-RA-00126R1**	1st Revision received:	June 12, 2023
	2nd Decision:	July 3, 2023 *accept with minor revision*
**TPC2023-RA-00126R2**	2nd Revision received:	July 6, 2023
	3rd Decision:	July 27, 2023 *acceptance pending, sent to science editor*
	Final acceptance:	Sept. 16, 2023

REPORT: (The report shows the major requests for revision and author responses. Minor comments for revision and miscellaneous correspondence are not included. The original format may not be reflected in this compilation, but the reviewer comments and author responses are not edited, except to correct minor typographical or spelling errors that could be a source of ambiguity.)

TPC2023-RA-00126 1st Editorial decision – *revision requested* Mar. 26, 2023

We have received reviews of your manuscript entitled "MADS8 stabilizes female reproductive development at high ambient temperature in barley and rice." Thank you for submitting your best work to The Plant Cell. The editorial board agrees that the work you describe is substantive, falls within the scope of the journal, and may become acceptable for publication, pending revision and potential re-review.

We ask you to pay attention to the following points in preparing your revision:

1) Further characterization of *mads8* mutant phenotype.

2) Clarify the temperature dependency related to the genetic redundancy of other E-class subclade genes. A comparison of amino acid sequence with other B- or C-class MADS genes, whose mutants show defects under normal growth conditions, would be informative. These non-responsive B- or C-class proteins should also be used in luciferase assay (Fig 6C).

3) More functional insight of the MADS proteins that distinguish the current work from your previously published study (i.e. Nature Plants, 2021).

--- Reviewer comments:

[Reviewer comments shown below along with author responses]

TPC2023-RA-00126R1 1st Revision received June 12, 2023

Reviewer comments and **author responses**:

<u>Reviewer #1:</u>

Overall comments: The study by Shen, Zhang et al. describes the role of barley and rice MADS8, a MADS-box transcriptional regulator, for female flower development. Authors convincingly show that loss of MADS8 (i.e. *mads8*) using CRISPR-lines results in temperature-dependent extra carpel formation. At high temperatures, *mads8* plants

often did not form ovules but instead continued carpel production during female reproductive development. Authors found that at high temperatures during early inflorescence development (W1-W6) transcript and protein levels of HvMADS8 were very similar; however, from W8 until W10 HvMADS8 was significantly up-regulated specifically for carpel and ovule development. Authors showed that HvMADS8 binding activity at high temperatures modulated floral development transcriptomic reprogramming (e.g. CUT&Tag data). In a dual-LUC assay authors further found that HvMADS8 directly regulates transcriptional expression of D-class genes, HvMADS13 and 21, at high temperatures. Altogether, authors conclude that MADS8 confers a new, conserved role by maintaining pistil number and ovule initiation in cereal crops.

I found this study of high scientific value, not only for the grass community. In fact, it has the potential to become a landmark paper by not only providing a deeper fundamental understanding of flower biology but similarly for its insights into temperature-related reproductive development in plants. And due to both aspects, it may gather lots of citations in the future! In its current form, I do not have major objections for it. However, I have listed a few points that, from my point of view, deserve more attention or clarifications:

Response to the overall comments: We greatly appreciate these positive comments.

Comment 1) L136-220 and Figure 1B: authors describe the pistil phenotype in much detail with excellent images and figures. However, authors did not mention anything about the ability to form grains in Hvmads8? In other words, can the mutant be easily maintained at ~15-22°C? Moreover, do the gray bars in figure 1B refer to potential future grains, or is grain set already affected below 25°C in Hvmads8? I assume 'no ovule - no grain" but did *Hvmads8* lines occasionally set grains even at 28°C? And if so, what was the grain set pattern along the spike? Randomly scattered, or position-dependent, e.g. only at the spike base? Please elaborate.

Response to comment #1: Thanks for this comment. We have added the data regarding the grain-forming ability of *hvmads8* and described that in the main text (please see Supplemental Figures S1C-S1F, page 7 lines 155-157 and lines 161-164). At lower temperatures (15°C-20°C), the *hvmads8* mutant can produce seeds with no noticeable differences in seed setting and morphology compared with WT (Supplemental Figure S1C, D). While at higher temperatures (25°C-28°C), the mutants could not produce any seeds, not even in the florets with a single pistil, as these pistils are fused with partial extra carpel and stigmas structures containing no ovules inside (please see Supplemental Figure S1E, F).

Comment 2) L362-379 and Figure 7B: please clarify similar points as for point 1).

Response to comment #2: We have also added new supplemental data and descriptions to address this (please see Supplemental Figure S4, page 16 lines 379-382 and 385-387). The *osmads8* rice mutant shows lower seed settings than WT at 28°C (please see Supplemental Figure S4A-B), although the pistil number and ovule structures display no obvious differences (Figure 7A-C). However, the shrunken grains of *osmads8* are indicative of roles of OsMADS8 in post-fertilization development, which might cause the lower seed setting rate of *osmads8* at 28°C (Supplemental Figure S4C-D). And at 34°C, the *osmads8* mutants also produce no seeds, similar to *hvmads8* mutants (Supplemental Figure S4E).

Comment 3) Title: the statement in the title appears slightly too cautious for my taste. I think authors could be a bit catchier and braver. For example, "MADS8 (non-italicized!) is indispensable for female reproductive development at high ambient temperatures in cereal crops".

Response to comment #3: We have changed it as suggested.

Comment 4) For the DISCUSSION: (a) I found it quite intriguing that the same pistil phenotype occurred in two mutants, two crops (barley, rice) but at different temperatures (28°C, 34°C). While at 28°C barley plants showed the phenotype, rice plants seemed to be happily growing at the same temperature?! Why? Could authors perhaps speculate about these species differences and what could cause them?

Response to comment #4a: We have explained the intrinsic difference between rice and barley in terms of optimal ambient temperature and have added our speculation in the discussion part (please see pages 21 lines 493-501).

(b) Along the same lines: would authors expect purifying selection for certain MADS8 haplotypes (i.e. protein variants) under higher temperatures, for example in the tropics? And if so, are rice MADS8 variants possibly more heat tolerant than variants from temperate cereals such as wheat, barley? Please let us know your opinion here.

Response to comment #4b: Thank you very much for raising this point. We have added the SNP information and discussed that in the discussion (please see page 21 lines 502-506, Supplemental Table S1). As MADS8 mainly functions to stabilize female reproductive development to cope with increased temperature, it is very likely that certain haplotypes grown at higher temperatures might have undergone purifying selections. However, the rice OsMADS8 protein is very conserved across varieties, as no SNP within coding regions of OsMADS8 was identified by us using the rice SNP-Seek Database derived from 3000 rice genomes (Wang et al., 2018). We then also investigated the *HvMADS8* coding region sequence across 267 barley genotypes acquired by exome sequencing (Supplemental Table S1) (Russell et al., 2016). In total six SNPs with low frequency were found, including four synonymous SNPs located at MADS and K domain, and two non-synonymous SNPs within the I domain. Further analysis of the two non-synonymous SNPs revealed a latitude range between 24.6-36.1 within the 5 barley accessions. Collectively, these SNP analyses revealed strong conservation of *MADS8* sequences. Although barley *HvMADS8* sequences contain more variations when compared with rice *OsMADS8* from our preliminary data, functional characterization of these SNPs, more evidence with larger database and also studies in other grass species will be needed to elucidate more on this issue.

Comment 5) M&M, L495-500: what was the genetic background of the *proHvMADS8:HvMADS8-eGFP* lines? Was the construct transformed into wt GP or in the CRISPR-mutant of *Hvmads8*? If in the mutant, please describe whether and how the phenotype was complemented using the eGFP-lines!

Response to comment #5: Thank you for this comment, we are sorry to have missed this information. The *proHvMADS8:HvMADS8-eGFP* construct was transformed into Golden Promise wild type. We have added this description in the method part in this revised version (please see page 23 lines 541-542).

Comment 6) REFs, L900+L903 are duplicated references, delete one.

Response to comment #6: Thanks for the comment. We have deleted one duplicated reference and changed the citation accordingly in the main text (page 15 line 359, page 20 line 461 and page 21 line 489).

Comment 7) I found it sometimes irritating that authors are not consistent while using the suitable notation for the gene or protein form. For example, already in the title MADS8 is italicized (indicating the gene!) even though, from my understanding, only proteins execute the function and are responsible for the phenotypic manifestation, not the transcripts/genes! The same applies to the following lines in which the protein form (non-italicized!) should be preferred: L037 (because the MADS8 protein maintains pistil number...), L097; L103; L222; L235; L248; L255 replace genes by proteins; L273; please cross-check for the remaining text (main and supplementary).

Response to comment #7: Many thanks for your critical comment. We are very sorry for the confusion and inconsistency of the gene or protein form. We have cross-checked the text and revised the following lines in this version: line 1; line 29; line 37; line 97; line 103; line 118; line 123; line 126; line 136; line 194; line 223; line 227; line 240; line 253; line 260; line 281; line 313; line 375; line 387; line 395; line 417; line 462; line 508.

Comment 8) L234 please add: ...by temperature during early development (W3-5).

Response to comment #8: We have added this description accordingly (please see page 10 line 239 in this revised manuscript).

<u>Reviewer 2:</u>

Overall comments: The manuscript by Shen et al. entitled "MADS8 stabilizes female reproductive development at high ambient temperature in barley and rice" demonstrates the molecular and biological function of a novel MADS-box transcription factor MADS8, which involves in maintaining pistil number and ovule initiation at high ambient temperature in barley and rice. The authors built a clear and convincing regulation network on how MADS8 is in control of floral organ development in response to high temperature, including reprogramming transcriptome expression profile and changing MADS8 binding activity of its downstream target genes. Additionally, the authors

revealed MADS8 regulates expression of D-class genes, MADS13 and MADS21, by directly binding to their promoter regions at high temperatures. These findings are interesting as it expands our knowledge on the mechanisms of floral organ development in response to high ambient temperature (or abiotic stress).

The manuscript itself is well written. Data analysis is sound and conclusions are well supported by the respective experiments. I have the following points for the authors to further improve the manuscript.

Response to the overall comments: We deeply appreciated these positive comments on our manuscript.

Comment 1) Figure 6F, right panel lane 1 - HvMADS8 is mislabelled.

Response to comment #1: Thank you very much for your comment, we apologize for the oversight and have corrected it in this revised manuscript.

Comment 2) Line 140 and 414: change "loss of function" to "loss-of-function".

Response to comment #2: Thanks for the comments, we have revised it accordingly (please see page 6 lines 140-141 and page 19 line 437).

Comment 3) Line 261: gene name should be in italic.

Response to comment #3: Thanks very much, we have corrected it in this revision (please see page 12 line 266).

Comment 4) Line 391: Figure 7 does not have panel E, correct it accordingly.

Response to comment #4: Thank you for the comment, we have changed it to Figure 7D in page 17 line 406-407 in this revised manuscript.

Comment 5) Line 536: change "2 µg" to "Two micrograms".

Response to comment #5: Thank you for the detailed comment, we have revised it as suggested (please see page 24 line 580).

Comment 6) Writing consistently either in British or American style (e.g., line 170 vs line 563).

Response to comment #6: Thanks for the comment, we have changed the spelling of center in lines 175; 205; 211; 217; 224 and 776, and we have also checked the rest of the text, made changes in lines 155; 228; 243; 491; 687; 784; 787 to keep it in American style.

<u>Reviewer 3:</u>

Overall comments: Shen et al. clearly describe barley and rice *SEPALLATA MADS8* genes are required to maintain floral meristem determinacy and ovule initiation at high temperatures. They created the loss-of-function *mads8* mutants in barley and rice by the CRISPR/Cas9 method, and both form multiple carpels that lack ovules only at high ambient temperatures. In *hvmads8*, two meristem-associated genes, HvDL and HvOSH1, are ectopically expressed at 28 °C, while two orthologs of D-class floral homeotic gene *HvMADS13* are downregulated. A CUT&Tag and ChIP-PCR analysis indicated that HvMADS8 binds to the promoters of targets stronger at higher temperatures. This paper is a continuous and extended work by the same group on a barley SEP protein, HvMADS1 controlling spike architecture at high temperatures through temperature-dependent binding to targets (Nature Plants 2021). Overall, this paper beautifully shows a conserved role for MADS8 in maintaining pistil number and ovule initiation in cereal crops at high temperatures and that temperature-responsive activity relies on increased binding to the targets. However, I have some serious reservations that I hope the authors can address. Below are several points the authors may want to consider.

Response to the overall comments: We greatly appreciate your comments very much.

Comment 1) Although this paper shows high temperature-dependent action of MADS1 in spikelet meristems is similarly applicable to MADS8 in floral meristems in barely and rice, the details of molecular mechanisms remain unclear. They showed that HvMADS8 RNA and protein levels do not change. The tetramer formation may not be

affected. Can they compare HvMADS1 and HvMADS8 with other MADS proteins in the E class and other classes (e.g., C class) whose mutants do not show temperature-dependency and discuss their functions at the levels of amino acid sequences?

Response to comment #1: Thank you very much for this comment. We have compared the amino acid sequence of all the five barley E-class proteins with B and C-class proteins including HvMADS3 that was previously proved without temperature-dependent gene activation by us (Li et al., 2021) (Please see Supplemental Figure S6 page 20-21 lines 479-485). However, more substantial information is needed to determine whether those MADS-box proteins act in a temperature-dependent manner. As to the E class family, we have acquired and examined the temperature-response of all the single mutants, demonstrating that the *hvmads5*, *hvmads34* and *hvmads7* single mutants do not show temperature-dependency phenotype. Surely, we cannot rule out the possibility that these proteins may have redundant functions with other members at higher temperatures. Besides, as shown in Response Figure 1, the MADS-domains, which is proposed to direct DNA bindings (Puranik et al., 2014; Huang et al., 2000), are very conserved across these MADS-box family proteins. While within the C-terminal domain, which is also thought to be involved in transcription activities (Callens et al., 2018; Muiño et al., 2014), varies dramatically between these proteins.

Comment 2) Related to comment 1), is temperature dependency related to the genetic redundancy of E-class subclade genes? In Fig 4, HvMADS8 appears to regulate some targets at 15°C. Phenotypic and molecular analyses in double-and multiple-mutants of the E-class genes are advisable.

Response to comment #2: Thanks very much for raising this point. We have discussed the functional redundancy of the two SEP3 family genes, *HvMADS8* and *HvMADS7*, in this revision (please see Supplemental Figure S5 page 18 lines 421-428). We have recently obtained the *hvmads7/hvmads8* double mutant, as the two genes belong to the same SEP3 subfamily and might function redundantly. The *hvmads7/hvmads8* double mutant phenotype is similar to the rice *lofsep* triple mutant (Wu et al., 2018), by showing spikelets with extra lemma and palea but without any inner floral organs. This phenotype suggested that HvMADS7 and HvMADS8 play redundant roles in specifying inner floral organ identities, a typical function among E-class proteins. However, as there is no pistil at the control temperatures for this double mutant, we can not determine the exact function of HvMADS7 in pistil number determinacy at higher temperatures. Thus, other genetic materials such as the complementary lines of *hvmads8* mutant using *HvMADS7* or other E-class members, will be needed to further elucidate the functional redundancy of other E-class proteins with HvMADS8 in temperature responsive female reproductive organ regulation.

Comment 3) Related to comment 1), how about protein modification of MADS8 under different temperatures?

Response to comment #3: We appreciate this comment. As the protein expression level of HvMADS8 is not affected by temperature (Figure 3F), protein modifications such as phosphorylation, glycosylation, acetylation and methylation etc. could all potentially play a role in HvMADS8 temperature responsive functions which could be an interesting point for our future research.

Comment 4) In Supplemental Fig 2, C-class genes were up-regulated at 28°C in *hvmads8*. In Arabidopsis, E-class genes are known to upregulate C-class genes. Please discuss their data based on previous knowledge.

Response to comment #4: Thank you for noting this, we have added this discussion in the text (please see page 12 lines 272-275). There are many MADS-box genes displaying altered expressions at *hvmads8* mutant, and it is known that complicated regulations exist within the MADS-box family members (Wu et al., 2018). Therefore, this up-regulation of C-class genes might be an indirect effect from the loss of HvMADS8, as the expression of *HvMADS1* also shows a significant increase at 28°C at the same W8.0 stage as the C-class genes do (please see Supplemental Figure S2I).

Comment 5) In Fig 6E, temperature-dependent increase of binding was observed at P1, but not at P3, on the HvMADS13 promoter. Is this increased binding of HvMADS8 protein responsible for *HvMADS13* regulation? What happens to the reporter line of *HvMADS13* with a mutation in P1?

Response to comment #5: Thank you for this comment. We have provided the new transient luciferase expression assay data in this manuscript to address this comment (please see Figure 6E, page 15 lines 347-349, page 28 lines 654-656). As suggested, we created a new *proHvMADS13MU::LUC* construct with mutated CArG-box sequences in the P1 binding site and perform the luciferase assay again with the original *proHvMADS13::LUC* construct, although

the activation of HvMADS8 on *HvMADS13* promoter still exists using this mutated construct, there is no significant difference between 20°C and 28°C. Our new data indicated that this P1 binding site contribute directly to the temperature-responsive gene activation of *HvMADS13* by HvMADS8. The new primers used for the construct are added in Supplemental Data Set S1.

Comment 6) In Fig 6C, other *HvMADS* genes, whose mutants do not show temperature-dependency, should also be used as controls for the *pro A-tract CArG-wt::LUC* reporter.

Response to comment #6: Thanks for this useful comment. In this revised manuscript, we have added HvMADS3 as a control as we have previously showed that HvMADS3 regulated gene expression independent of temperature (Li et al., 2021) , and we have added this new information accordingly in the main text and method (please see Figure 6C, page 15 lines 337-338, page 27 line 649). The new primers used for the construct are added in Supplemental Data Set S1.

Cited references:

Callens, C., Tucker, M.R., Zhang, D., and Wilson, Z.A. (2018). Dissecting the role of MADS-box genes in monocot floral development and diversity. J. Exp. Bot. 69: 2435–2459.

Cortijo, S., Charoensawan, V., Brestovitsky, A., Buning, R., Ravarani, C., Rhodes, D., van Noort, J., Jaeger, K.E., and Wigge, P.A. (2017). Transcriptional Regulation of the Ambient Temperature Response by H2A.Z Nucleosomes and HSF1 Transcription Factors in Arabidopsis. Mol. Plant 10: 1258–1273.

Huang, K., Louis, J.M., Donaldson, L., Lim, F.L., Sharrocks, A.D., and Clore, G.M. (2000). Solution structure of the MEF2A-DNA complex: Structural basis for the modulation of DNA bending and specificity by MADS-box transcription factors. EMBO J. 19: 2615–2628.

Li, G., Kuijer, H.N.J., Yang, X., Liu, H., Shen, C., Shi, J., Betts, N., Tucker, M.R., Liang, W., Waugh, R., Burton, R.A., and Zhang, D. (2021). MADS1 maintains barley spike morphology at high ambient temperatures. Nat. Plants 7: 1093–1107.

Muiño, J.M., Smaczniak, C., Angenent, G.C., Kaufmann, K., and Van Dijk, A.D.J. (2014). Structural determinants of DNA recognition by plant MADS-domain transcription factors. Nucleic Acids Res. 42: 2138–2146.

Perrella, G., Bäurle, I., and van Zanten, M. (2022). Epigenetic regulation of thermomorphogenesis and heat stress tolerance. New Phytol. 234: 1144–1160.

Perrella, G., Zioutopoulou, A., Headland, L.R., and Kaiserli, E. (2020). The impact of light and temperature on chromatin organization and plant adaptation. J. Exp. Bot. 71: 5247–5255.

Puranik, S. et al. (2014). Structural basis for the oligomerization of the MADS domain transcription factor SEPALLATA3 in Arabidopsis. Plant Cell 26: 3603–3615.

Russell, J. et al. (2016). Exome sequencing of geographically diverse barley landraces and wild relatives gives insights into environmental adaptation. Nat. Genet. 48: 1024–1030.

Vanwallendael, A., Soltani, A., Emery, N.C., Peixoto, M.M., Olsen, J., and Lowry, D.B. (2019). A Molecular View of Plant Local Adaptation: Incorporating Stress-Response Networks. Annu. Rev. Plant Biol. 70: 559–583.

Wang, W. et al. (2018). Genomic variation in 3,010 diverse accessions of Asian cultivated rice. Nature 557: 43–49.

Wu, D., Liang, W., Zhu, W., Chen, M., Ferrándiz, C., Burton, R.A., Dreni, L., and Zhang, D. (2018). Loss of LOFSEP transcription factor function converts spikelet to leaf-like structures in rice. Plant Physiol. 176: 1646–1664.

TPC2023-RA-00126R1 2nd Editorial decision – *accept with minor revision* July 3, 2023

We have received reviews of your manuscript entitled "MADS8 is indispensable for female reproductive development at high ambient temperatures in cereal crops." On the basis of the advice received, the board of reviewing editors

would like to accept your manuscript for publication in The Plant Cell. This acceptance is contingent on revision based on the comments of our reviewers. In particular, please consider the following:

Please clarify the relationship between temperature dependence and genetic redundancy of E-class genes. In particular, whether HvMADS7 single mutant shows temperature-dependent phenotypes.

TPC2023-RA-00126R2 2nd Revision received July 6, 2023

Reviewer comments and **author responses**:

Reviewer #1:

I am very happy with the authors' responses, and how they addressed my previous points. For the newly revised sentences please replace "seed" by "grain".

Response: As per the suggestion, we have changed "seed" to "grain" on page 7 line 157.

Reviewer 3:

The authors almost fully addressed my concerns. It is a pity that the relationship between temperature dependence and genetic redundancy of E-class genes remains unknown. It would still be informative to describe whether HvMADS7 single mutant shows temperature-dependent phenotypes.

Response: Thank you very much for this comment. As per the suggestion, we have included the genotype and phenotype data of the *hvmads7* single mutants at both control and high temperatures and discussed the phenotype in this revised manuscript (Please see Supplemental Figure S5 A-B, page 18 lines 422-424). The *hvmads7* single mutants display no detectable differences in pistil morphology at both 15°C and 28°C. Integrating the phenotypes of the three SEP3 family mutants together (Figure 1 and Supplemental Figure S5), it is suggested that HvMADS8 and HvMADS7 have a conserved redundant function in regulating inner floral organ identities at control temperatures (15°C), while HvMADS8 is more indispensable for pistil development at higher temperatures (28°C).

TPC2023-RA-00126R2 3rd Editorial decision – *acceptance pending* July 27, 2023

We are pleased to inform you that your paper entitled "MADS8 is indispensable for female reproductive development at high ambient temperatures in cereal crops" has been accepted for publication in The Plant Cell, pending a final editorial review by a science editor.

Final acceptance from Science Editor Sept. 16, 2023
